# The Transfer Hydrogenation of Cinnamaldehyde Using Homogeneous Cobalt(II) and Nickel(II) (E)-1-(Pyridin-2-yl)-N-(3-(triethoxysilyl)propyl)methanimine and the Complexes Anchored on Fe_3_O_4_ Support as Pre-Catalysts: An Experimental and In Silico Approach

**DOI:** 10.3390/molecules28020659

**Published:** 2023-01-09

**Authors:** Fortunate P. Sejie, Olayinka A. Oyetunji, James Darkwa, Isaac N. Beas, Banothile C. E. Makhubela, Nelson Y. Dzade, Nora H. de Leeuw

**Affiliations:** 1 Department of Chemistry, University of Botswana, Gaborone Private Bag UB 00704, Botswana; 2 Botswana Institute for Technology Research and Innovation, Gaborone Private Bag 0082, Botswana; 3 Department of Chemical Sciences, University of Johannesburg, P.O. Box 524, Auckland Park 2006, South Africa; 4 Department of Energy and Mineral Engineering, Pennsylvania State University, University Park, PA 16802, USA; 5 School of Chemistry, Cardiff University, Main Building, Park Place, Cardiff CF10 3AT, UK; 6 School of Chemistry, University of Leeds, Leeds LS2 9JT, UK

**Keywords:** Fe_3_O_4_-immobilised pre-catalysts, transfer hydrogenation, cinnamaldehyde

## Abstract

The imino pyridine Schiff base cobalt(II) and nickel(II) complexes (**C1** and **C2**) and their functionalised γ-Fe_3_O_4_ counterparts (**Fe_3_O_4_@C1** and **Fe_3_O_4_@C2**) were synthesised and characterised using IR, elemental analysis, and ESI-MS for **C1** and **C2**, and single crystal X-ray diffraction for **C1**, while the functionalised materials **Fe_3_O_4_@C1** and **Fe_3_O_4_@C2** were characterized using IR, XRD, SEM, TEM, EDS, ICP-OES, XPS and TGA. Complexes **C1**, **C2** and the functionalised materials **Fe_3_O_4_@C1** and **Fe_3_O_4_@C2** were tested as catalysts for the selective transfer hydrogenation of cinnamaldehyde and all four pre-catalysts showed excellent catalytic activity. Complexes **C1** and **C2** acted as homogeneous catalysts with high selectivity towards the formation of hydrocinnamaldehyde (88.7% and 92.6%, respectively) while **Fe_3_O_4_@C1** and **Fe_3_O_4_@C2** acted as heterogeneous catalysts with high selectivity towards cinnamyl alcohol (89.7% and 87.7%, respectively). Through in silico studies of the adsorption energies, we were able to account for the different products formed using the homogeneous and the heterogeneous catalysts which we attribute to the preferred interaction of the C=C moiety in the substrate with the Ni centre in **C2** (−0.79 eV) rather than the C=O (−0.58 eV).

## 1. Introduction

The difficulty in separating products from homogeneously catalyzed hydrogenation reactions is a major disadvantage that limits the use of homogeneous catalysts for industrial production, despite their often-high catalytic activities compared to heterogeneous catalysts [1]. The use of water-soluble catalysts and supported catalysts are some of the strategies employed towards improving the recyclability of homogeneous catalysts. Supported catalysts have gained scientific interest as they are intermediates between homogeneous and heterogeneous catalysts [2]. Catalytic systems with a ligand bearing the alkoxy(alkyl)silane moiety offer potential for attaching a solid support to the catalytic system through covalent bonding between the hydroxyl moiety of the support and the alkoxide groups on the ligand [3], which provides sustainable and recyclable active catalysts, as the support improves product isolation and recyclability of the catalysts without the use of solvents [4]. The structure of supported catalysts dictates their activity, and it is hence vital that we study the structure and reactivity of the unsupported catalyst in comparison with the supported catalysts to gain better understanding of the activity, selectivity, and other effects of the support [2]. 

**Scheme 1:**
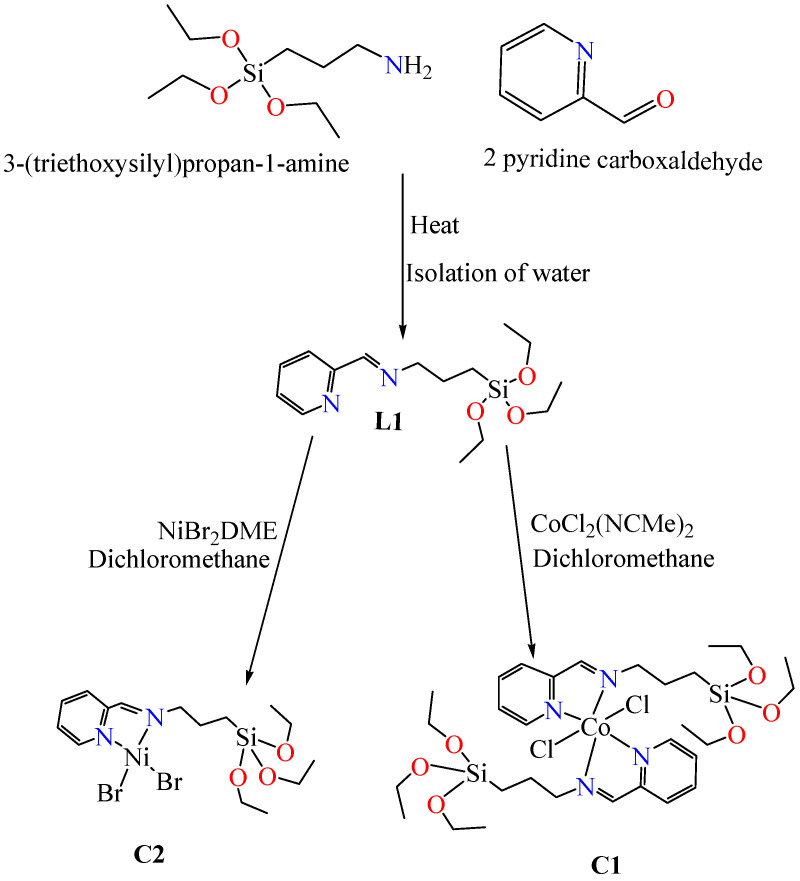
Synthesis of **L1** and complexes **C1** and **C2**.

The catalytic hydrogenation of organic compounds possessing multiple unsaturated bonds, such as α, β-unsaturated aldehydes using both homogeneous and heterogeneous catalysts is particularly challenging, which hence requires active catalytic sites that can discriminate between the closely related moieties [5,6]. Cinnamaldehyde (CAL) is an example of an α, β-unsaturated aldehyde which can be converted to valuable chemical intermediates by hydrogenation of either the olefinic or the carbonyl moieties using homogeneous catalysts. Owing to the presence of different chemical moieties in varying chemical environments, it is a better subject for a comparative study on the catalyst activity and selectivity than other substrates such as furfural [7]. Chemoselective hydrogenation of cinnamaldehyde leads to the formation of either hydro-cinnamaldehyde (HCAL), cinnamyl alcohol (COL), or hydro-cinnamyl alcohol (HCOL) (Scheme 1) [6]. HCOL and COL are important intermediates in the production of cosmetics, fragrances and flavourings, while HCAL is used as an intermediate in the production of drugs used for HIV treatment [7,8,9]. The reduction of the unsaturated moieties in cinnamaldehyde by hydrogen molecules proceeds through the 1,2 or 1,4 addition pathways (Scheme 1). Since the 1970s, supported nickel [10], palladium [11,12], and cobalt [13] catalysts have been shown to hydrogenate olefins [14]. Supported cobalt [14,15,16] catalysts in particular are known to be selective in the hydrogenation of the olefinic bond in subtrates that contain both the olefinic and carbonyl bonds [16]. 

In view of the above, it is critically important to determine the structure of molecular compounds that are used as catalysts, especially if such compounds have bidentate ligands that could render these compounds polymeric, in order to establish how such compounds act as catalysts [17,18]. There are only limited reports in the literature on the use of supported catalysts in the hydrogenation process and comparing their catalytic activity and selectivity to their homogeneous counterparts, either computationally (in silico) or by experimental methods, is therefore highly relevant for progress in this field.

The aim of this study was to employ unsupported and supported catalysts for hydrogenation reactions, and the use of in silico methods to elucidate the selectivity of the supported catalyst in comparison with their homogeneous analogues. As such, this report first presents the synthesis and characterisation of homogeneous Co^2+^ and Ni^2+^ N-(1-(pyridin-2-yl)ethylidene)-3-(triethoxysilyl)-1-propanamine pre-catalysts and their γ-Fe_3_O_4_ immobilized counterparts, and the application of all four pre-catalysts in the transfer hydrogenation of cinnamaldehyde using formic acid as a hydrogen source. 

## 2. Results and Discussion 

### 2.1. Syntheses and Characterization of the Ligand and Homogeneous Pre-Catalysts 

Complexes **C1** and **C2** were prepared from the reaction of (N-(1-(pyridin-2-yl)ethylidene)-3-(triethoxysilyl)-1-propanamine (**L1**) with the appropriate metal salts (Scheme 1). Characterization of **L1** and **C2**, which are known compounds [19], can be found in Figures S1–S3; whilst **C1**, which is new, was characterized as described below. Complex **C1** was isolated as an orange solid that is soluble in dichloromethane and methanol. The IR spectrum showed the following peaks which are assigned to the stretching frequencies in parenthesis; 2970 cm^−1^ (ν_C-N_), 2886 cm^−1^ (ν_C-C_),1645 cm^−1^ (ν_C=N_), 1456 cm^−1^ (ν_C-C_),1365 cm^−1^(ν_C-C_), 1074 cm^−1^(ν_Si-O_),950 cm^−1^(ν_C-O_). When the IR peaks of **L1** (Figure S4) are compared to those of pre-catalysts **C1** and **C2**, there is a shift in the stretching frequency of the imine bond from 1649 cm^−1^ to (1646, and 1645) cm^−1^. The observed decrease in the stretching energy is associated with the pseudo-single bond that the imino bond assumes as the ligand interacts with the metal centre [20], which requires less stretching energy.

#### Crystal Structure Determination of **C1**

**Figure 1:**
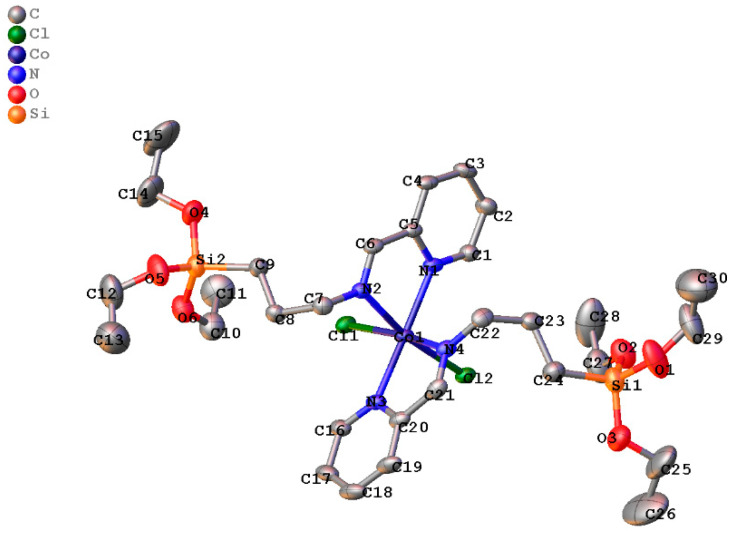
Molecular structure of **C1** with thermal ellipsoids. Hydrogen atoms are omitted for clarity.

The crystal structure of **C1** is shown in Figure 1. It is mononuclear with two chelating ligands bound to the metal in a bidentate fashion via the nitrogen atoms. The crystal structure is different from those observed for bidentate cobalt complexes that have bidentate nitrogen chelating bipyridyl and imidazole ligands, which show polymeric crystal structures [17]. Table 1a shows the metal complex to have a monoclinic crystal structure. In the solid-state structure shown in Figure 1, the interaction between the cobalt metal centres with the nitrogen donor atoms is evident. The interaction between the metals and the free imine is weaker (longer bond lengths [Table 1b]) compared to that of the aromatic imine (shorter bond lengths [Table 1b]). **C1** crystalizes in a centrosymmetric crystal system (monoclinic [Table 1a]), with a primitive lattice (P) allowing a 2-fold/180° rotation. Table 1b shows shorter bond lengths between the pyridyl nitrogen and the metal centre compared to the imine nitrogen, suggesting stronger interactions of the cobalt with the pyridyl nitrogen, and the bond angles for an octahedral geometry. Table 1b shows the bite angle of (78.86 and 78.80)° between the cobalt atom and the nitrogen ligand, which shows distortion from the ideal bite angle of 90° for an octahedral geometry. 

### 2.2. Characterization of Magnetite and the Supported Pre-Catalysts

**Scheme 2:**
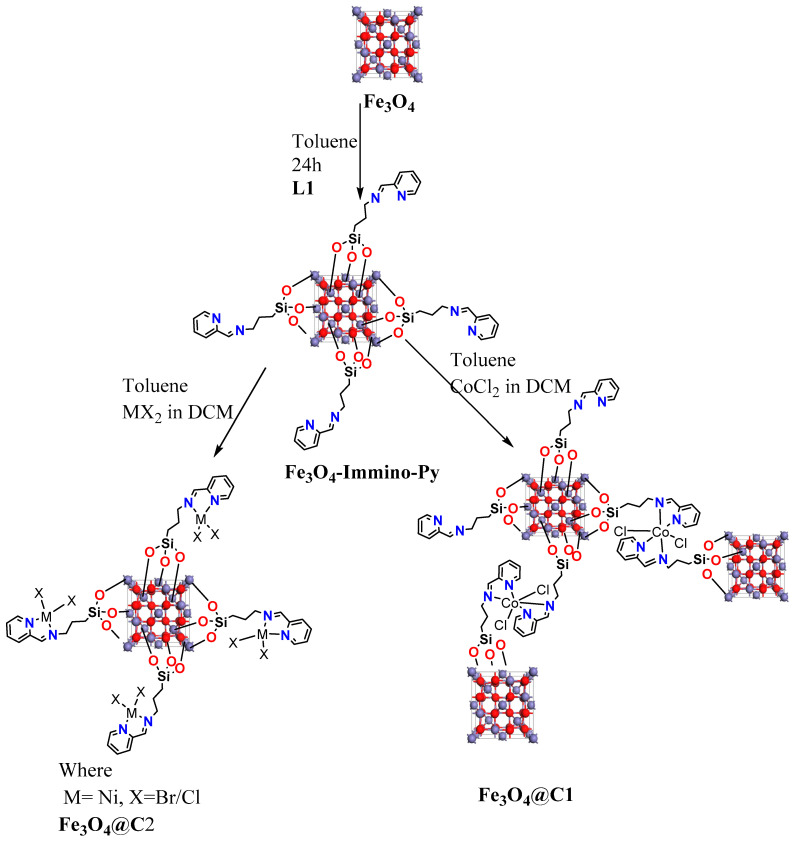
Synthesis of **Fe_3_O_4_@C1** and **Fe_3_O_4_@C2** catalysts.

The general reaction schemes that produced the two supported pre-catalysts, **Fe_3_O_4_@C1** and **Fe_3_O_4_@C2**, are shown in Scheme 2. Complexes **C1** and **C2** were immobilized on superparamagnetic iron oxide (Fe_3_O_4_) nanoparticles according to previously reported procedures [3,21,22] with some modifications. Figure S5 shows the IR, TEM, SEM-EDS, XRD and particle distribution of Fe_3_O_4_-immino-py. From Figure S5, PXRD patterns show that the inverse spinel phase of magnetite is preserved, as its characteristic peaks are observed, but the phases at hkl values 220 and 422 appear broad, which is an indication of non-uniform macro-straining on the magnetite lattice as the ligand is attached. The IR spectra show the appearance of a new peak at 1700 cm^−1^, which is attributed to the presence of the imine moiety in the immobilised ligand [23]. The TEM micrographs show the agglomerated spherical crystalline particles with average particle size of 3.4–4 nm, whereas the SEM image shows an inhomogeneous surface with agglomerated particles. The EDS provides evidence for the presence of Si and nitrogen in addition to magnetite atoms (Fe and O). These results show successful anchoring of the **L1** onto magnetite.

**Figure 2:**
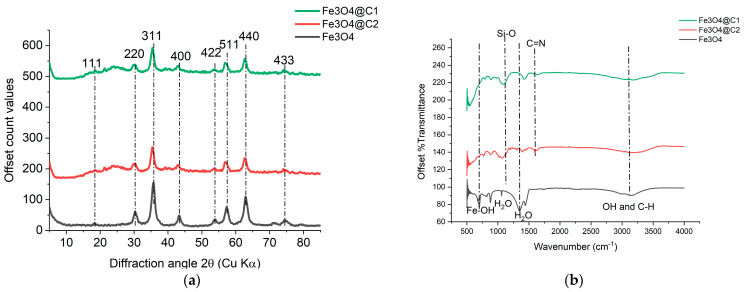
Powder X-rays diffraction patterns (**a**) and FTIR spectra (**b**) of magnetite (Fe_3_O_4_), **Fe_3_O_4_@C2** and **Fe_3_O_4_@C1**.

Figure 2a shows the powder X-ray diffractions of as-synthesised magnetite. The peaks indexed as the X-ray diffractions from the following planes: (220), (311), (400), (422), (511), and (440), correspond to a face centred cubic inverse spinel phase of magnetite [20,21]. The phases at hkl values 220 and 422 appear broad for the system with supported pre-catalysts. The crystallite size measurements of the supported pre-catalysts were determined from the full width at half maximum (FWHM) of the strongest reflection of the hkl diffraction peak, using the Debye-Scherrer formula, $D=\frac{k\lambda }{\beta Cos\theta }$ where D is the crystallite mean size and a shape factor k = 0.9 is used, λ is the wavelength of the radiation, β the full width at half maximum (FWHM) in radians in the 2θ scale, and θ = $\frac{2\theta }{2}$ in radians. The average crystal sizes calculated for the pre-catalysts were 3.40 ± 0.40, 3.0 ± 0.5, and 4.0 ± 0.3 nm for magnetite, **Fe_3_O_4_@C1**, and **Fe_3_O_4_@C2**, respectively. Figure 2b shows the IR spectra of magnetite, **Fe_3_O_4_@C1**, and **Fe_3_O_4_@C2**, with the Fe-O-Fe stretches at 702 cm^−1^. The peaks at 1359 cm^−1,^ and 1443 cm^−1^ correspond to the CH_2_ stretch and the adsorbed H_2_O stretch is observed at 1060 cm^−1^, while the hydroxy moiety (ν_OH_) is observed at 3287 cm^−1^. The IR of the supported pre-catalysts is staggered in Figure 2b. The stretching frequencies of Fe-O-Si appear at higher stretching frequencies, 769 and 887 cm^−1^, compared to 702 cm^−1^ for the Fe-O functionality. This shift to higher frequencies shows that a higher energy is required to cause a stretch in the Fe-O bond in the supported pre-catalyst, due to the attached heavy atom (Si), which is also evident from the disappearance of a sharp narrow peak at 1060 cm^−1^. The imino functionality of the ligand (C=N) is detected at 1599 cm^−1^. The appearance of the stretching frequency at 1018 cm^−1^ corresponds to the presence of the Si-O bond.

The Ni and Co metal loadings on the pre-catalysts have been determined by Inductively Coupled Plasma Optical Emission Spectroscopy (ICP-OES) to be 1.22 and 1.37 m/m% for the pre-catalyst **Fe_3_O_4_@C1** and **Fe_3_O_4_@C2**, respectively, which are comparable to literature reports [3,22]. These observations further support the successful coordination of the active metal to the immobilised ligand. The effect of attaching a ligand/pre-catalyst to magnetite causes strain onto the lattice, which can be identified from the shifts or broadening of the peaks. Peak broadening is a characteristic of non-uniform macro-strain on the lattice, while peak shift is a result of uniform micro-strain. From Figure 2a, the peaks broaden with minor shifts as the pre-catalysts are supported onto the support. The resulting total strain was estimated from the Williamson-Hall (W-H) equation (Equation (1)), where the micro-strain (ε) was obtained from the slope of the straight-line fit, where D is the crystallite mean size, and a shape factor k = 0.9 is used, λ is the wavelength of the radiation, β the full width at half maximum (FWHM) in radians in the 2θ scale, and θ = $\frac{2\theta }{2}$ in radians.

(1) $$\beta \cos\theta =\frac{K}{D}+\varepsilon \cdot 4\mathrm{Sin}\theta$$

(2) $$\varepsilon =\frac{\mathrm{Slope}}{4\mathrm{Sin}\theta }$$

**Figure 3:**
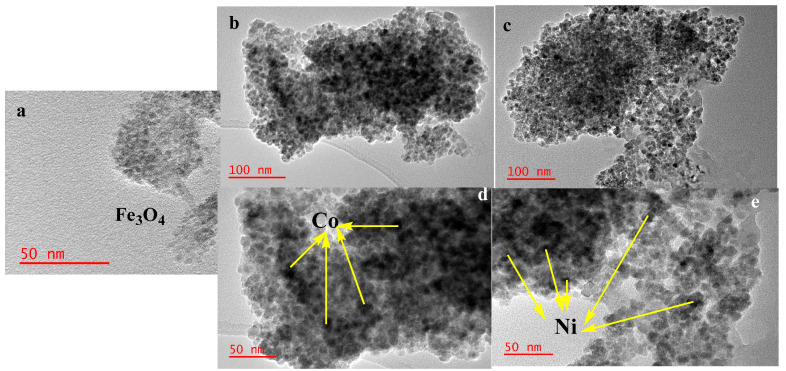
TEM micrograph of **Fe_3_O_4_** (**a**), **Fe_3_O_4_@C1** (**b**,**d**) **Fe_3_O_4_@C2** (**c**,**e**), respectively.

The macro-strains estimated from the W-H plot are shown in Table S1 alongside the metal loading determined from ICP-OES. Attaching the ligand to magnetite exerts a lattice strain (ε) of 9.8 × 10^−3^, giving a highly strained lattice. However, coordinating the metal centre to the immobilised ligand leads to a decrease in the lattice strain of magnetite from 6.9 × 10^−3^ and 9.8 × 10^−3^ to 7.0 × 10^−3^ for **Fe_3_O_4_@C1** and **Fe_3_O_4_@C2**, respectively, which indicates that the immobilised pre-catalysts are more stable compared to the ligand only, because of lower lattice stain. In this study, the heterogenized catalysts were subjected to thermal analysis in the temperature range of 40–1000 °C. Figure S6 shows the total % weight loss of 10, 40 and 60% for **Fe_3_O_4_**, **Fe_3_O_4_@C1** and **Fe_3_O_4_@C2**, respectively. Between 0 to 400 °C, **Fe_3_O_4_** shows a mass loss of 8% which corresponds to loss of the adsorbed water molecules, while for **Fe_3_O_4_@C2**, and **Fe_3_O_4_@C1**, mass losses of 40 and 60% are observed, respectively, between 400–1000 °C, which correspond to the loss of the anchored catalyst. These results are similar to the observations made by Sobhani et al. for a palladium-Schiff base complex immobilized covalently on magnetic nanoparticles [1]. Transmission electron microscopy was used to determine the average particle size, morphology and relative shape of magnetite. Figure 3 shows TEM micrographs of magnetite **Fe_3_O_4_** (a), compared to **Fe_3_O_4_@C1** (b and d) and **Fe_3_O_4_@C2** (c and e). From the TEM micrographs, the nano-catalysts are agglomerated into spherical shapes. This tendency is not surprising, given the nanoparticles’ small size and magnetic properties [24]. The appearance of dark shades in the images (b–e) implies that the electron beam did not penetrate the sample, which could be a result of the homogeneous phase of the catalyst that is attached to the support occluding the electron beam. In contrast, these dark shades are not apparent in Figure 3a showing the pure magnetite.

**Figure 4:**
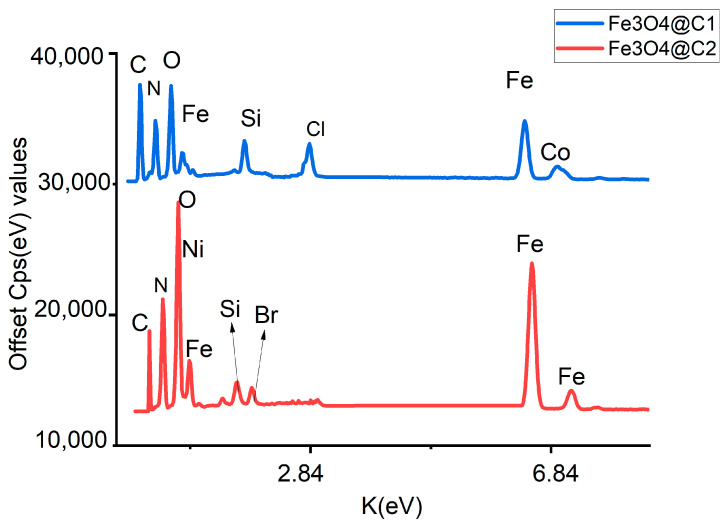
The EDS spectra of **Fe_3_O_4_@C1** and **Fe_3_O_4_@C2** nanoparticles.

The SEM images show inhomogeneous surfaces with non-uniform particle shapes (Figure S7) for magnetite, **Fe_3_O_4_@C1**, and **Fe_3_O_4_@C2**, which is not surprising due to the magnetic nature of the particle, which is often difficult to overcome even by solvent dispersion. The corresponding EDS spectra of **Fe_3_O_4_@C1** and **Fe_3_O_4_@C2** are shown in Figure 4, which reveal the presence of the metals (Ni and Co) in the supported pre-catalysts, showing that the active metal centre was successfully coordinated to the supported ligand [25]. 

**Figure 5:**
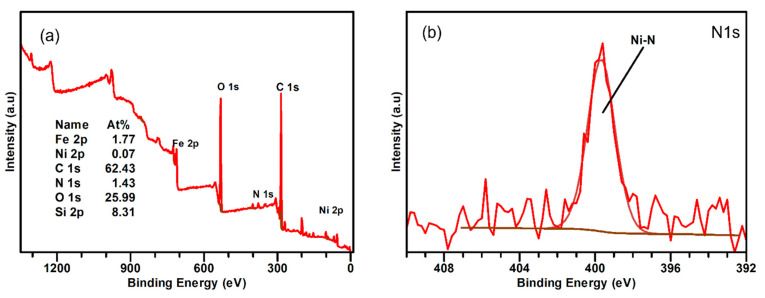

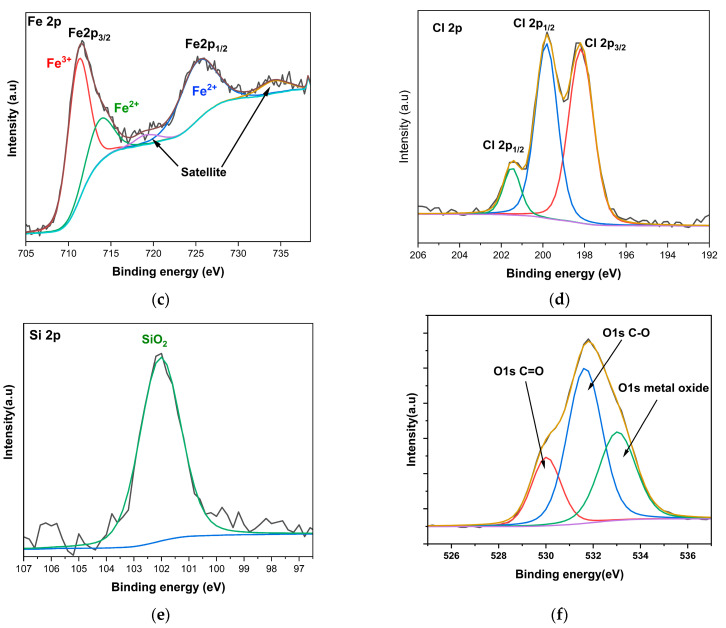
(**a**) Survey of **Fe_3_O_4_@C2** catalyst, (**b**) The N 1s high-resolution spectrum, and analysis for: Fe2p (**c**), Cl2p (**d**), Si2p (**e**) and (**f**) O1s elements in **Fe_3_O_4_@C2** catalyst.

Figure 5a shows the overview spectra of the composition of the catalyst **Fe_2_O_3_@C2** with atomic percentage, whereas the analysis for the elements Fe, Cl, Si are shown in Figure 5c–e. We can fit the N 1s spectrum, Figure 5b, into a typical peak corresponding to a metal-N species (399.1 eV); these species in Ni-N-C support the dispersion of Ni in the form of Ni-N coordination [1,24].

**Figure 6:**
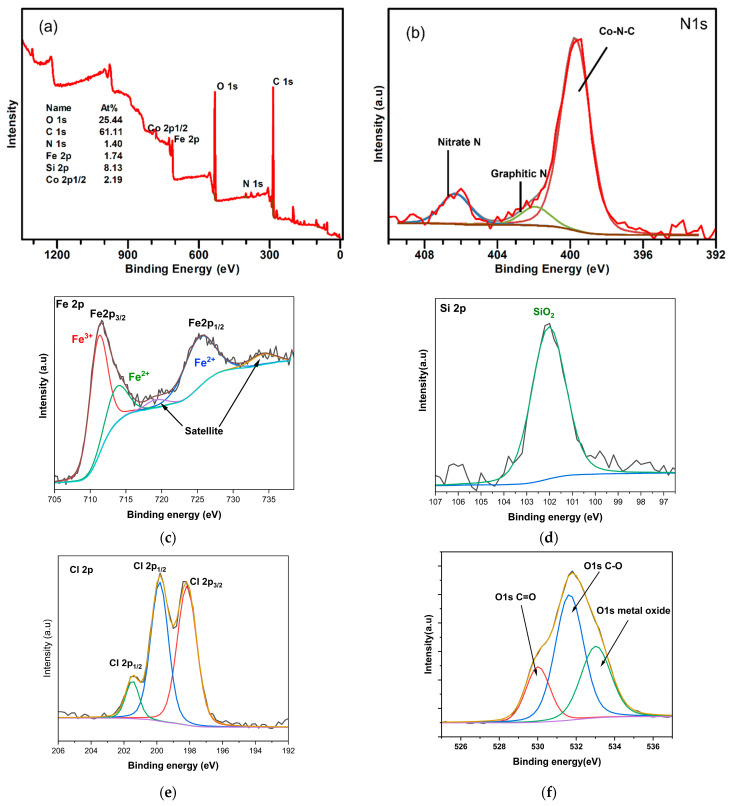
(**a**) Survey of **Fe_3_O_4_@C1** catalyst, (**b**) The N 1s high-resolution spectrum and analysis for Fe2p (**c**), Si 2p (**d**), Cl2p (**e**) and O1s (**f**) elements in **Fe_3_O_4_@C1** catalyst.

Figure 6a–e, shows the XPS survey and high resolution analysis of the N, Si, Fe, Cl and O in pre-catalyst **Fe_2_O_3_@C1**. The bonding configurations of the N 1s spectrum can be divided into three distinct peaks at 399.2 eV, 401.9 eV and 406.3 eV, corresponding to Co-N, graphite-N, and nitrate-N species, respectively [26,27,28]. The nitrogen atoms are mainly in the form of Co-N (79.76%), nitrate-N (12.47%) and graphite-N (7.76%) [26,27]. The N 1s bond with the Co metal may be the possible active site in the reactions [28,29,30]. In both Figure 5c and Figure 6c, the major peak at 710.8 eV (red curve) confirms the characteristic peak from Fe2p3/2 core level electrons that can be attributed to Fe^3+^ octahedral and tetrahedral sites, while Fe^2+^ species are found at binding energy 714.1 eV (green curve). Furthermore, the peak at 723.9 eV (blue curve) confirms Fe^2+^ species in octahedral sites of the Fe_3_O_4_ [31,32].

### 2.3. Catalytic Evaluation

**Scheme 3:**
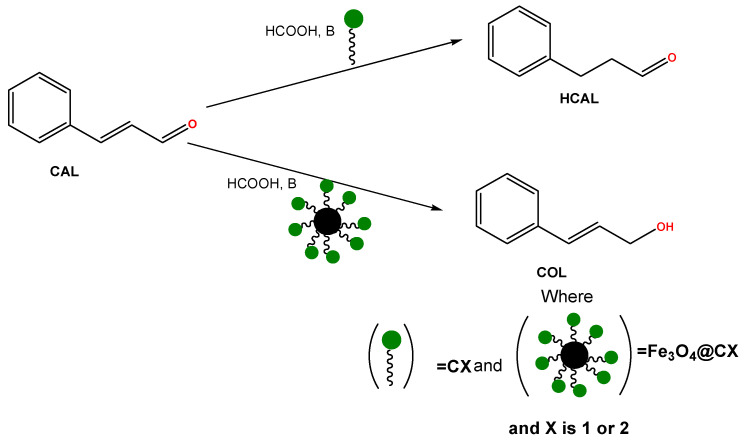
General scheme for the transfer hydrogenation of cinnamaldehyde to hydro cinnamaldehyde and cinnamyl alcohol using the synthesised catalyst.

We have investigated the transfer hydrogenation of cinnamaldehyde to different products as shown in Scheme 3. The examples of the ^1^NMR and GC spectra used to estimate the conversions are shown in Figures S8–S11. 

#### 2.3.1. Effect of Temperature on Catalyst Activity and Selectivity of **C1**, **C2**, **Fe_3_O_4_@C1** and **Fe_3_O_4_@C2**

Temperature was established to be an influential factor in the transfer hydrogenation process. For **C2**, Table 2 entries 1–5 show that a gradual temperature increase from 40 °C to 120 °C (Table 2) resulted in a corresponding gradual increase in the percentage conversion from 0% at 40 °C to 89.9% at 120 °C, while the selectivity towards the hydro-cinnamaldehyde product also gradually increased over the same temperature rise. The same observation is made for **C1** (Table 2, entries 6–8), where an increase in the temperature from 80 to 120 °C increased the conversion from 38.5% to 48.8%. **C2** showed higher conversions of cinnamaldehyde compared to **C1** (Table 2, entries 5 and 8). Both homogeneous pre-catalysts showed high selectivity towards hydro-cinnamaldehyde in the range of 85–92%, while HCOL production is in the range of 7.4–13.1% for both **C1** and **C2** (Table 2, entries 1–6). These results show that the olefinic moiety (C=C) was selectively reduced compared to the carbonyl moiety (C=O) by the **C1** and **C2** catalysts. 

Magnetite has been reported to have catalytic activity for processes such as the degradation of organic compounds [33], whereas it is reported to be inert towards other catalytic processes [21]. As shown in Table 2, entries 9–13, an increase in the temperature also gave better conversions for both **Fe_3_O_4_@C1** and **Fe_3_O_4_@C2**. However, the selectivity changed from high yields of HCAL to high yields of COL for the reactions catalysed by both **Fe_3_O_4_@C1** (89.7%) and **Fe_3_O_4_@C2** (87.7%). Entry 14, Table 2 shows that no conversion was observed when using pure magnetite as a catalyst. From this study, it is evident that the changes in the selectivity from HCAL to COL as the catalysts are immobilized on magnetite are due to the surface effects of the support. The supported catalysts reduce the substrates through a different mechanism compared to the homogeneous catalysts, which is a possible cause of the varying selectivities observed. Similar selectivity observations have been reported for the hydrogenation of ketones and aldehydes using catalysts supported on magnetite [34]. The selectivity of catalysts for the α,β unsaturated aldehyde is of interest, and cinnamaldehyde with its conjugated phenyl ring has been reported to be reduced to corresponding alcohols over heterogeneous catalysts [35]. Moreover, the use of Fe is also reported to be a known promoter of the hydrogenation of C=O in α,β unsaturated aldehydes, as observed in a study of crotonaldehyde and cinnamaldehyde over supported Pt, Ru, and Rh catalysts, with Fe(II) added as a promoter [35,36]. The homogeneous catalysts convert cinnamaldehyde to hydro-cinnamaldehyde, followed by further conversion to hydrocinnamyl alcohol. With the supported homogeneous catalysts, cinnamaldehyde is converted to both cinnamyl alcohol and hydro-cinnamaldehyde but further conversion of these two products to a fully saturated system (hydrocinnamyl alcohol) is not observed for the heterogeneous catalysts. 

#### 2.3.2. Catalyst Reusability Studies

##### Recyclability of Pre-Catalysts **C1**, **C2**, **Fe_3_O_4_@C1** and **Fe_3_O_4_@C2**

**Figure 7:**
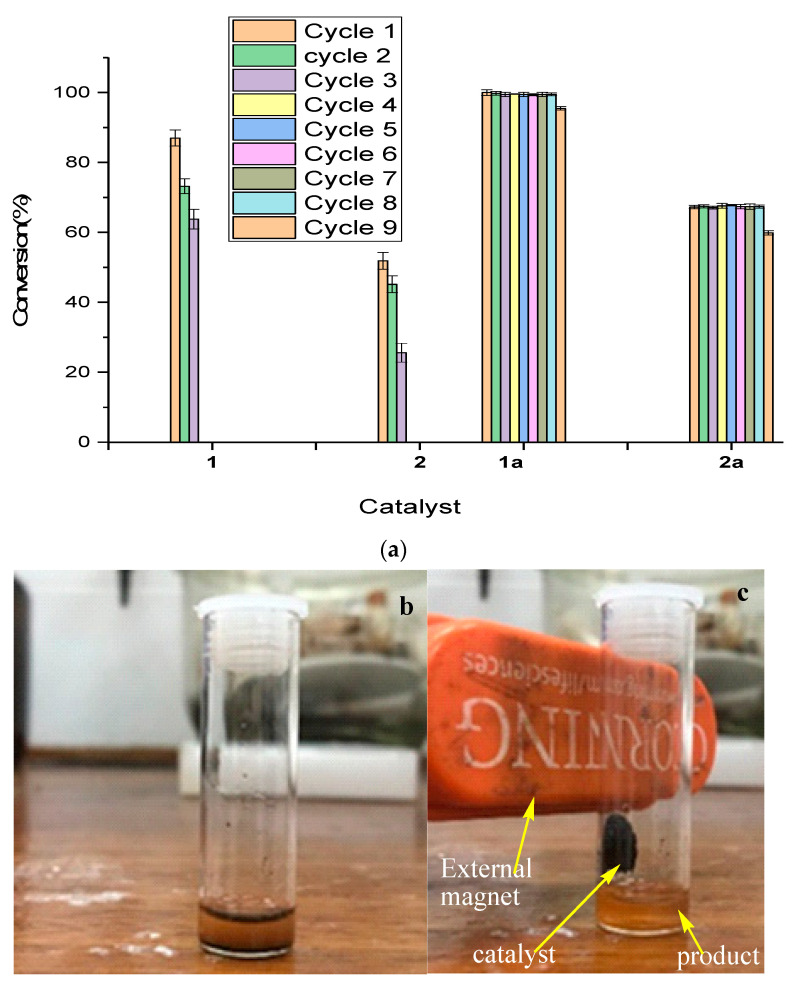
Recyclability studies using pre-catalyst **C1** and **C2** (labelled **1** & **2**), **Fe_3_O_4_@C2** (**1a**) and **Fe_3_O_4_@C1** (**2a**). Reaction conditions: For pre-catalyst **C1** and **C2**: CAL (20 mmol), (catalyst:0.1 mol% for 1 & 2 and 0.8 mol% for **Fe_3_O_4_@C2** and **Fe_3_O_4_@C1** (formic acid 40 mmol, base 20 mmol),120 °C, 24 h Conversions estimated by Gas chromatography. (**a**) shows the maximum number of cycles the homogeneous catalyses before deactivation while (**b**,**c**) shows the isolation of the spent catalyst from the reaction mixture.

It is important to establish the number of cycles over which a catalyst can convert a substrate to products before deactivation. In this study, the homogeneous catalysts were recycled as per methods reported in the literature [37]. In the experiment, the reaction was simultaneously repeated with catalyst recovery from the reaction mixture. After the first catalytic cycle, the products were extracted with hexane and diethyl ether. This allowed the pre-catalysts to precipitate, before being dried under reduced pressure and weighed. Dichloromethane was used to wash the catalyst back into the autoclave reactor. The reactor was then charged with the pre-catalyst, substrate, base, and formic acid. For **Fe_3_O_4_@C1** and **Fe_3_O_4_@C2**, after the first cycle the catalyst was separated from the products using a magnet (Figure 7b,c) and washed with dichloromethane and oven dried. The reactor was then charged with the substrate, base, and formic acid. Recyclability studies of homogeneous catalysts are rare in the literature, especially for **C1** and **C2**. From Figure 7, the homogeneous catalysts are recyclable up to 3 cycles, giving conversions ranging between 50–38% and 80–70% from cycle 1 to cycle 3 for catalysts **C1** and **C2**, respectively. The loss of activity in the third cycle could be due to catalyst poisoning/fouling by the organic waste or catalyst loss due to workup. The supported catalysts could be used up to 8 cycles, (Figure 7a) giving conversions of 65% and ~99% for **Fe_3_O_4_@C1** and **Fe_3_O_4_@C2**, respectively, maintained over 8 cycles. Loss of activity starting in cycle 9 is observed for both **Fe_3_O_4_@C1** and **Fe_3_O_4_@C2**, which is attributed to the same causes as the homogeneous catalysts.

##### Characterization of the Used **Fe_3_O_4_@C1** and **Fe_3_O_4_@C2**

To investigate the stability of the used catalysts, we characterized the materials by TEM, IR, XRD and EDS, and the results were compared to those of the fresh catalysts. The TEM images in Figure S12a,b show no changes in the morphology and size of the particles, whereas the functional groups and elements of the supported ligand are still observed in the IR (Figure S12c) and EDS (Figure S13) spectra. From Figure S14, the inverse spinel of magnetite is still preserved after 9 catalytic cycles. The XPS survey of **Fe_3_O_4_@C2** (Figure S15) and **Fe_3_O_4_@C1** (Figure S17) and the high-resolution analysis of N, C, Si, Cl/Br, O, Fe in **Fe_3_O_4_@C2** (Figure S16) and **Fe_3_O_4_@C1** (Figure S18) show no significant changes in the catalysts after use.

#### 2.3.3. Metal Leaching and Homogeneity Tests

##### Homogeneity Studies for Pre-Catalysts **C1** and **C2**

Mercury interacts with nanoparticles to form amalgam (Hg_y_M_x_), which completely blocks the heterogeneous active sites, leading to inhibited catalytic activity due to heterogeneous species that might have formed in the reaction mixture as a result of catalyst decomposition [38]. Formation of amalgam leads to over 50% decrease in the conversions for heterogeneously catalysed reactions. From Figure S19, it is evident that there was a small decrease in the conversions from 48.7% to 40.7% for pre-catalyst **C1** and 89.5% to 83.6% for pre-catalyst **C2**. These decreases are, however, not enough to account for the possibility of Ni^2+^ and Co^2+^ decomposition, and the mercury poisoning test has been reported to often lead to inaccurate conclusions with M(II) pre-catalysts, especially in the case of dynamic catalytic systems [38,39].

##### Leaching Test for Pre-Catalyst **Fe_3_O_4_@C1** and **Fe_3_O_4_@C2**

The leaching of Co and Ni from the **Fe_3_O_4_@C1** and **Fe_3_O_4_@C2** catalysts was determined using methods reported in the literature [40]. After a 24 h catalytic reaction time, the catalyst was removed from the reaction mixture using a magnet. The reaction mixture was placed in a crucible and heated to 500 °C under a ramping regime of 10 °C/min, before being calcined at 600 °C for 3 h. The residue was weighed and digested in aqua regia and analyzed for Ni and Co using ICP-OES. The results showed that 0.4 and 0.5 mg/kg of Co and Ni, respectively, were detected in the residue, i.e., less than 1% of the initial catalyst loadings, which indicates that only negligible amounts of Ni and Co were leached from the surface of magnetite to the solution. As such, we may infer that the catalysts are stable and tolerate the reaction conditions for the transfer hydrogenation of cinnamaldehyde. 

#### 2.3.4. In Silico Studies of the Selectivity of the Catalysts

**Figure 8:**
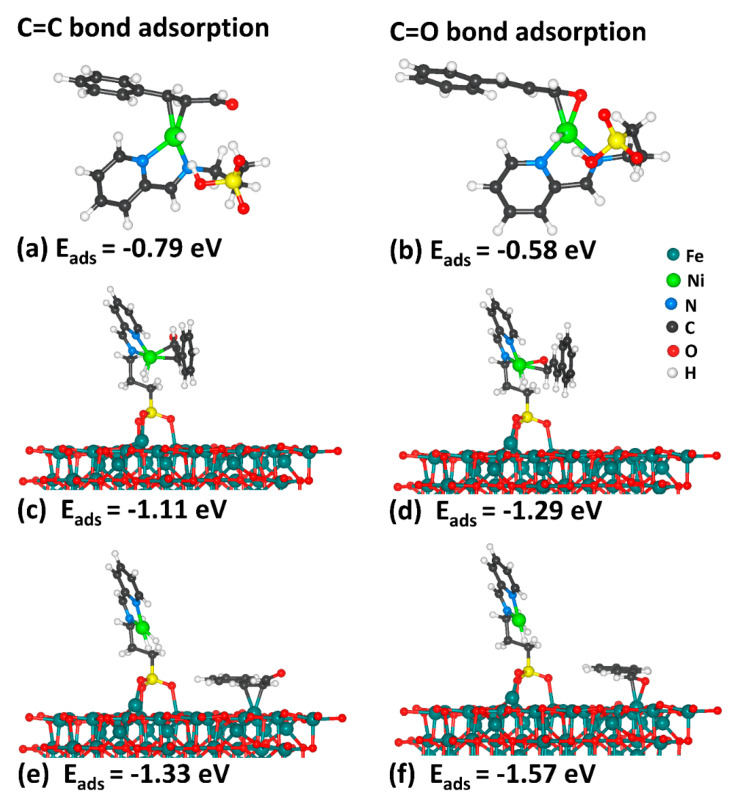
Optimized adsorption geometries and energetics (E_ads_) of CAL on the homogeneous catalyst via (**a**) C=C bond and (**b**) C=O bond; on the heterogeneous catalyst via C=C bond (**c**,**e**) and C=O bond (**d**,**f**) at the Ni site and on the magnetite support.

In supported catalysts, the active metal is dispersed on the surface of the support, which in turn can alter the catalytic activity and selectivity because of the active metal-support interactions. To gain detailed insight into the selectivity of the homogenous and heterogeneous catalysts towards the C=C and C=O bonds in cinnamaldehyde, we have carried out calculations based on the density functional theory (DFT) to predict the lowest energy adsorption geometries and the adsorption energetics (Figure 8). The active species formed through oxidative addition of the hydrogen molecule to the metal centre in the pre-catalysts were used to investigate favourable interactions between the Ni metal and the olefinic or carbonyl moieties present in cinnamaldehyde.

The DFT calculations reveal that C=C bond adsorption to the homogenous catalyst is more stable (E_ads_ = −0.79 eV) than that of the C=O bond (−0.58 eV), indicating a higher selectivity to HCAL. In contrast, at the heterogeneous catalyst, the adsorption of CAL via a C=O bond at both the Ni site (−1.29 eV) and on the magnetite support (−1.57 eV) is energetically more favourable than via the C=C bond at the Ni site (−1.11 eV) and on the support (−1.33 eV). These results indicate that the heterogeneous catalysts favour the C=O centred adsorption and hydrogenation, which results in its selectivity towards the formation of COL rather than towards HCOL. These results are consistent with the experimental results, which show that the homogenous catalysts have higher HCAL selectivity, while the heterogeneous catalysts produce higher COL selectivity.

## 3. Materials and Methods

All chemicals were purchased from Sigma Aldrich and were used without further purification. All solvents were dried in molecular sieves 24 h before use. E)-1-(pyridin-2-yl)-N-(3-(triethoxysilyl)propyl) methanimine (**L1**) and (E)-1-(pyridin-2-yl)-N-(3-(triethoxysilyl)propyl) methanimino nickel bromide (**C2**) were prepared according to the literature procedures [25]. All hydrogenation reactions were performed in a PPV-CTRO1-CE high-pressure reactor vessel fitted into a high-pressure autoclave reactor with in-built stirring, heating, and cooling systems.

### 3.1. Characterization Techniques

Nuclear magnetic resonance (NMR) spectra were recorded on a Bruker Ultrashield-400 MHz spectrometer (^1^H: 400 MHz, ^13^C{^1^H}: 100 MHz) in chloroform using tetramethylsilane (TMS) as a reference. All the chemical shifts were reported in ppm (δ) using TMS as a reference. The functional groups were confirmed using a Thermo Nicolet IR instrument fitted with an ATR probe. Mass spectrometry was carried out on a Water Synapt G2 electrospray ionization mass spectrometer in the negative or positive-ion mode. Elemental analyses were done using a Thermo Scientific Flash 2000 Series CHNO elemental analyzer. 

Powder X-ray diffraction (PXRD) data were collected using an XPERT-PRO diffractometer (Cu K_α_ radiation) with a current flow of 40 mA and voltage of 40 kV. Single crystal X-ray data were collected using a Bruker KAPPA APEX II DUO diffractometer with graphite monochromator Mo–K_α_ radiation (l ¼ 0.71073 A). The data collection was carried out at low temperatures (173 K) using a Cryostream cooler. Unit cell refinement and data reduction were performed using the program OLEX. Structure solutions and refinement were achieved by the SHELXS program. 

For TEM analysis, the supported catalysts were dispersed in ethanol followed by ultrasonication for 15 min. The samples were placed on a carbon-coated copper grid, dried at room temperature. The transmission electron microscope images were collected using a JEOL Model JEM 2100F electron transmission electron microscopy (TEM) system operating at a voltage of 200 kV. For the scanning electron microscopy (SEM), the heterogeneous catalysts were coated with carbon, and images were collected using the Tescan Vega 3LMH scanning electron microscope coupled with energy-dispersive X-ray spectroscopy (EDS) system. The Co and Ni metal loading on **Fe_3_O_4_@C2** and **Fe_3_O_4_@C1** were determined using Spectro Arcos ICP-OES.

The XPS analyses were performed with an ESCAlab 250Xi instrument. The X-ray source was chosen as the monochromatic Al K line (1486.7 eV). XPS can detect all elements except hydrogen and helium, it probes the sample surface to a depth of 2–10 nm, and has detection limits of around 0.1 at%. The pass energy was 20 eV, and the energy increment was 100 meV. Narrow scan photoelectron spectra were recorded (between four and eight scans). The spectra were charge-corrected to the main line of the carbon 1s spectrum (adventitious carbon) set at 284.8 eV. Quantifying the detected elements and deconvolution of thespectra was performed with the CasaXPS software (version 2.3.25). 

### 3.2. Synthesis of (E)-1-(Pyridin-2-yl)-N-(3-(triethoxysilyl)propyl) Methanimino Cobalt Chloride (**C1**)

Complex **C1** was prepared by adding a solution **L1** (0.311 g, 1.0 mmol) in dry dichloromethane (10 mL) to a solution of [Co(NCME)_2_Cl_2_] (1.0 mmol) in dry dichloromethane (20 mL) under nitrogen. The reaction mixture was stirred for 24 h at room temperature. After completion of the reaction, the volume of solution was reduced to about 5 mL on a rotary evaporator and solid product was precipitated by adding ice-cold diethyl ether (15 mL). The product was filtered, washed with 3 parts of diethyl ether (10 mL) and dried in vacuum. Elemental Analysis (found values are in parenthesis): C, 40.92% (39.33%); H, 5.95% (5.55%); N, 6.36% (6.22%).

### 3.3. Synthesis of the Supported Catalysts **Fe_3_O_4_@C1** and **Fe_3_O_4_@C2**

The immobilized catalysts were synthesized by a modification of a method reported in the literature. 0.3 g Fe_3_O_4_ in 30 mL of toluene was added a solution of 0.3 g of **L1** in 5 mL of dichloromethane. The mixture was then reacted together under ultra-sonication for 0.5 h. The metal precursor (2 mmol) was further added to the reaction mixture and allowed to react for 24 h. The reaction mixture was centrifuged, and the solvent discarded. The solid product was washed with dichloromethane and dried in air. 

### 3.4. Catalytic Selectivity and Activity Tests

#### Transfer Hydrogenations Studies

A Teflon reactor (50 mL) was used in this study. A mixture of the catalysts, cinnamaldehyde, formic acid, and a base was heated to the desired temperature after purging four times with nitrogen gas at a stirring speed of 960 rpm for a desired reaction time. At the end of the reaction, the reactor vessel was cooled, followed by the release of the excess gas. A sample of the mixture was then analysed by ^1^H NMR spectroscopy and/or gas chromatography to determine the conversion and product selectivity. Examples of the typical spectrum are shown in Figures S7 and S8 (^1^H NMR) and the GC shown in Figures S9 and S10.The conversion of cinnamaldehyde and selectivity of the products were calculated using the following equations:

(3) $$\mathrm{CAL}\;\mathrm{conv}=\left(\frac{\mathrm{Initial}\;\mathrm{moles}\;\mathrm{of}\;\mathrm{CAL}-\mathrm{Final}\;\mathrm{moles}\;\mathrm{of}\;\mathrm{CAL}}{\mathrm{initial}\;\mathrm{moles}\;\mathrm{of}\;\mathrm{CAL}}\right)\times 100$$

(4) $$\mathrm{HCAL}\;\mathrm{selectivity}=\left(\frac{\mathrm{moles}\;\mathrm{of}\;\mathrm{HCAL}}{\mathrm{Total}\;\mathrm{moles}\;\mathrm{of}\;\mathrm{products}}\right)\times 100$$

(5) $$\mathrm{HCOL}\;\mathrm{selectivity}=\left(\frac{\mathrm{moles}\;\mathrm{of}\;\mathrm{HCOL}}{\mathrm{Total}\;\mathrm{moles}\;\mathrm{of}\;\mathrm{products}}\right)\times 100$$

(6) $$\mathrm{COL}\;\mathrm{selectivity}=\left(\frac{\mathrm{moles}\;\mathrm{of}\;\mathrm{COL}}{\mathrm{Total}\;\mathrm{moles}\;\mathrm{of}\;\mathrm{products}}\right)\times 100$$

### 3.5. Computational Details 

The spin-polarized density functional theory (DFT) calculations were carried out using the Vienna Ab Initio Simulation Package (VASP) [41,42,43,44] wherein the interactions between the core and valence electrons were treated using the Project Augmented Wave (PAW) method [41]. The exchange-correlation potential was calculated using the Perdew-Burke-Ernzerhof (PBE) generalized gradient approximation (GGA) functional [42] with a Hubbard correction (PBE+U), which accounts for the Coulomb interaction of localized *d*-electrons [43,44]. In this study, the value for the on-site Coulomb interaction term for Fe was set to U_eff_ = 3.7 eV, which has been demonstrated to provide sufficiently accurate lattice parameters, electronic, and magnetic properties of Fe_3_O_4_ [45]. Long-range dispersion interactions were accounted for using the Grimme DFT-D3 scheme [46]. The Kohn-Sham wave functions were expanded in a plane-wave basis set with a kinetic energy cut-off of 500 eV. Structural optimizations were conducted using the conjugate-gradient algorithm until the residual Hellmann–Feynman forces on all relaxed atoms reached 0.01 eV/Å.

The bulk Fe_3_O_4_ material was modelled in the full conventional cubic unit cell of 56 atoms, where a 5 × 5 × 5 Monkhorst–Pack [47] k-point mesh was used to sample the Brillouin zone. The most stable (001) surface was employed for the construction of the supported pre-catalysts and for the adsorption characterization of CAL. A vacuum region of 20 Å was added to the c-axis to avoid interactions between periodic slabs. Different coordination modes of CAL (C=C and C=O bond adsorption) were explored to find the most stable adsorption configurations [48,49]. No symmetry constraints were imposed in any of the calculations on the structural optimization of the CAL-catalyst systems, and in particular, the CAL was free to move away laterally and vertically from the initial binding site(s) or reorient itself to find the lowest-energy adsorption configuration. The strength of adsorption for CAL was determined by calculating the adsorption energy (E_ads_) as follows:

E_ads_ = E_catalyst+CAL_ − (E_catalyst_ + E_CAL_) (7)

where E_catalyst+CAL_, E_catalyst_, and E_CAL_ are the relaxed total energies of the catalyst-CAL systems, the isolated catalyst, and the CAL adsorbate molecule, respectively. The adsorption is deemed exothermic and favourable if the calculated adsorption energy is negative. 

## 4. Conclusions

We have reported the synthesis and characterisation of the homogeneous cobalt(II) and nickel(II) pre-catalysts (**C1** and **C2**) of the E-1pyridyl-N-3-triethoxysilylpropyl methanimine ligand and their magnetite-immobilised counterparts **Fe_3_O_4_@C1** and **Fe_3_O_4_@C2**, as efficient catalysts in the transfer hydrogenation of cinnamaldehyde. Catalyst **Fe_3_O_4_@C1** and **Fe_3_O_4_@C2** are efficient, recyclable, and easily recoverable from the reaction mixture, yielding over 80% of cinnamyl alcohol with a metal loading of 1.37%m/m and 1.22%m/m, respectively, while the homogeneous catalysts yielded over 89% of hydro-cinnamaldehyde. Supporting the homogeneous catalysts on magnetite was found to alter the selectivity from the reduction of C=C to the reduction of C=O, which change in selectivity was established to be associated with the interaction of the favoured C=O moiety at the support surface, as compared to the C=C. The catalysts **Fe_3_O_4_@C1** and **Fe_3_O_4_@C2** show negligible metal leaching at less than 1% after 9 cycles, which creates less pollution and more stable catalysts. Thus, anchoring the homogeneous catalysts to a support is an effective way to improve catalyst recyclability, but as observed in this work, product selectivity can be influenced by the magnetite support, even though the support itself is catalytically inactive in the transfer hydrogenation process. 

## Figures and Tables

**Table 1:** (**a**) Crystal data and structure refinement for **C1**. (**b**) Selected bond Lengths and bond angles for **C1**.

(**a**)						
**Identification Code**					**C1**	
Empirical formula					C_30_H_52_Cl_2_CoN_4_O_6_Si_2_	
Formula weight					750.76	
Temperature/K					150.00(10)	
Crystal system					monoclinic	
Space group					P2_1_/c	
a/Å					19.0910(7)	
b/Å					12.7328(4)	
c/Å					17.5010(6)	
α/°					90	
β/°					115.307(5)	
γ/°					90	
Volume/Å^3^					3845.9(3)	
Z					4	
ρ_calc_mg/mm^3^					1.297	
μ/mm^−1^					0.69	
F(000)					1588	
2Θ range for data collection					5.15 to 61.856°	
Index ranges					−26 ≤ h ≤ 26, −17 ≤ k ≤ 16, −23 ≤ l ≤ 23	
Reflections collected					65540	
Independent reflections					8989[R(int) = 0.1308]	
Data/restraints/parameters					8989/0/406	
Goodness-of-fit on F^2^					0.969	
Final R indexes [I >= 2σ (I)]					R_1_ = 0.0391, wR_2_ = 0.0799	
Final R indexes [all data]					R_1_ = 0.0642, wR_2_ = 0.0871	
Largest diff. peak/hole/e Å^−3^					0.43/−0.31	
(**b**)						
**Atom**	**Atom**	**Length/Å**	**Atom**	**Atom**	**Atom**	**Angle/˚**
Co1	N1	2.1808(12)	N1	Co1	N2	76.82(5)
Co1	N2	2.1332(12)	N1	Co1	N3	169.36(5)
Co1	N4	2.1361(12)	N3	Co1	N4	76.80(5)
Co1	N3	2.1660(13)				

**Table 2:** Catalytic evaluation of the catalysts for transfer hydrogenation of cinnamaldehyde at varying temperature using all the pre-catalysts.

Entry	Cat	Temp (°C)	Conv (%)	TOF	Selectivity (%)		
					HCAL	COL	HCOL
1	**C2**	40	0.0	0	0.0	0.0	0.0
2	**C2**	60	49.1	20.45	87.3	0.0	12.7
3	**C2**	80	75.3	31.37	87.1	0.0	12.9
4	**C2**	100	82.9	34.54	86.9	0.0	13.1
5	**C2**	120	89.9	37.45	92.6	0.0	7.40
6	**C1**	80	38.5	16.04	91.9	0.0	8.11
7	**C1**	100	43.3	18.04	89.8	0.0	10.2
8	**C1**	120	48.8	20.33	88.7	0.0	11.3
9 *	**Fe_3_O_4_@C2**	100	86.5	4.282	12.9	87.1	0.0
10 *	**Fe_3_O_4_@C2**	120	99.8	4.940	12.3	87.7	0.0
11 *	**Fe_3_O_4_@C2**	140	100	4.950	11.9	88.1	0.0
12 *	**Fe_3_O_4_@C1**	120	68.7	3.454	10.2	89.7	0.0
13 *	**Fe_3_O_4_@C1**	140	73.9	3.848	9.1	90.9	0.0
14	**Fe_3_O_4_**	120	0.0	0	0.0	0.0	0.0

Reaction conditions: CAL (20 mmol), (catalyst 0.1 mol% (formic acid 40 mmol, base 20 mmol),120 °C, 24 h Conversions estimated by gas chromatography (Figure S8. * 0.8% moles of the catalysts. Error estimates ±0.69 (**C1**), ±0.91 (**C2**), ±0.14 (**Fe_3_O_4_@C2**) and ±0.58 (**Fe_3_O_4_@C1**).

## Data Availability

The data supporting this investigation are available from the corresponding authors upon request.

## Supplementary Materials

The following supporting information can be downloaded at: https://www.mdpi.com/article/10.3390/molecules28020659/s1, FigureS1: ^1^H NMR spectra of (E)-1-(pyridin-2-yl)-N-(3-(triethoxysilyl)propyl)methanimine, Figure S2: The ^13^C NMR spectra of 2:(E)-1-(pyridin-2-yl)-N-(3-(triethoxysilyl)propyl)methanimine, Figure S3: Time of Flight Electrospray Ionisation spectrum of **C2** in negative mode, Figure S4: The IR spectra of the ligand and the corresponding homogeneous catalysts, Figure S5: P-XRD, FTIR, SEM, EDS, particle size distribution and TEM characterization of Fe_3_O_4_-immino-py, Figure S6: Thermal gravimetric analysis of the supported pre-catalysts, Figure S7: The SEM images of the fresh heterogeneous catalysts, Figure S8: Example of the ^1^H NMR spectrum of the isolated products of CAL hydrogenation using homogeneous catalyst **C2** after 16 h of reaction time, Figure S9: Example of the ^1^H NMR spectrum of the crude mixture after CAL hydrogenation using Fe_3_O_4_@**C2** after catalyst 8 h of reaction time, Figure S10: Example of the GC spectrum of CAL hydrogenation using homogeneous catalyst **C2**, Figure S11: Example of the GC spectrum of CAL hydrogenation using homogeneous **Fe_3_O_4_@C2** catalyst, Figure S12: The TEM images of catalyst **Fe_3_O_4_@C2** (a) and **Fe_3_O_4_@C1** (b) and the IR analysis(c) of after the 9th cycle, Figure S13: The EDS spectra of the spent catalysts, Figure S14: The XRD of the spent catalysts, Figure S15: The XPS survey of spent **Fe_3_O_4_@C2**, Figure S16: The high-resolution analysis of the elements detected in **Fe_3_O_4_@C2**, Figure S17: The XPS survey of spent **Fe_3_O_4_@C1**, Figure S18: The high-resolution analysis of the elements detected in **Fe_3_O_4_@C1**, Figure S19: Homogeneity tests for the transfer hydrogenation of CAL using catalysts **C1** and **C2** in the presence of liquid mercury. Reaction conditions: CAL (20 mmol), catalyst (0.02 mmol) formic acid (40 mmol), base (20 mmol), 120 °C, 2 mg Hg (0), 24 h. Conversions are estimated by gas chromatography. Table S1: Calculated strain on the lattice of magnetite for the supported pre-catalysts, ligand, and the support.

## References

[1] Sobhani S., Ramezani Z. (2016). Synthesis of Arylphosphonates Catalyzed by Pd-Imino-Py-γ-Fe_2_O_3_ as a New Magnetically Recyclable Heterogeneous Catalyst in Pure Water without Requiring Any Additive. RSC Adv..

[2] Michalska Z.M., Webster D.E. (1975). Supported Homogeneous Catalysts. Chem. Tech..

[3] Sobhani S., Falatooni Z.M., Asadi S., Honarmand M. (2016). Palladium-Schiff Base Complex Immobilized Covalently on Magnetic Nanoparticles as an Efficient and Recyclable Catalyst for Heck and Suzuki Cross-Coupling Reactions. Catal. Lett..

[4] Jiang F., Cai J., Liu B., Xu Y., Liu X. (2016). Particle Size Effects in the Selective Hydrogenation of Cinnamaldehyde over Supported Palladium Catalysts. RSC Adv..

[5] Ide M.S., Hao B., Neurock M., Davis R.J. (2012). Mechanistic Insights on the Hydrogenation of α,β-Unsaturated Ketones and Aldehydes to Unsaturated Alcohols over Metal Catalysts. ACS Catal..

[6] Liu H., Li Z., Li Y. (2015). Chemoselective Hydrogenation of Cinnamaldehyde over a Pt-Lewis Acid Collaborative Catalyst under Ambient Conditions. Ind. Eng. Chem. Res..

[7] López-Linares F., Gonzalez M.G., Páez D.E. (1999). The Regioselective Biphasic Hydrogenation of Trans-Cinnaldehyde by Meta Sulfonatophenyl-Diphenylphosphine (TPPMS) Ru(II) and Os(II) Species. The Influence of Ionic Strength, Ligand Tensoactivity and Metal Nature in the Selective Production of the Unsatura. J. Mol. Catal. A Chem..

[8] Chen Z., Chen J., Li Y. (2017). Metal–Organic-Framework-Based Catalysts for Hydrogenation Reactions. Chin. J. Catal..

[9] Kostas I.D., Antonopoulou G., Potamitis C., Raptopoulou C.P., Psycharis V. (2017). Platinum Complexes with a Methoxy-Amino Phosphine or a Nitrogen-Containing Bis(Phosphine) Ligand. Synthesis, Characterization and Application to Hydrogenation of Trans-Cinnamaldehyde. J. Organomet. Chem..

[10] Zhang L., Chen X., Peng Z., Liang C. (2018). Chemoselective Hydrogenation of Cinnamaldehyde over MOFs-Derived M2Si@C (M = Fe, Co, Ni) Silicides Catalysts. Mol. Catal..

[11] Bhogeswararao S., Srinivas D. (2010). Chemoselective Hydrogenation of Cinnamaldehyde over Pd/CeO_2_-ZrO_2_ Catalysts. Catal. Lett..

[12] Li R., Yao W., Jin Y., Jia W., Chen X., Chen J., Zheng J., Hu Y., Han D., Zhao J. (2018). Selective Hydrogenation of the C=C Bond in Cinnamaldehyde over an Ultra-Small Pd-Ag Alloy Catalyst. Chem. Eng. J..

[13] Wu B., Huang H., Yang J., Zheng N., Fu G. (2012). Selective Hydrogenation of α,β-Unsaturated Aldehydes Catalyzed by Amine-Capped Platinum-Cobalt Nanocrystals. Angew. Chem. Int. Ed..

[14] Chai M., Liu X., Li L., Pei G., Ren Y., Su Y., Cheng H., Wang A., Zhang T. (2017). SiO_2_-Supported Au-Ni Bimetallic Catalyst for the Selective Hydrogenation of Acetylene. Cuihua Xuebao/Chin. J. Catal..

[15] Lv X., Zhang L., Xing F., Lin H. (2016). Controlled Synthesis of Monodispersed Mesoporous Silica Nanoparticles: Particle Size Tuning and Formation Mechanism Investigation. Microporous Mesoporous Mater..

[16] Leng F., Gerber I.C., Axet M.R., Serp P. (2018). Selectivity Shifts in Hydrogenation of Cinnamaldehyde on Electron-Deficient Ruthenium Nanoparticles. C. R. Chim..

[17] Feng X., Chen J.L., Bai R.F., Wang L.Y., Wei J.T., Chen X.X. (2016). Two Unique Cobalt-Organic Frameworks Based on Substituted Imidazole-Dicarboxylate and Dipyridyl-Type Ancillary Ligands: Crystal Structures and Magnetic Properties. Inorg. Chem. Commun..

[18] Feng X., Liu J., Li J., Ma L.F., Wang L.Y., Ng S.W., Qin G.Z. (2015). Series of Coordination Polymers Based on 4-(5-Sulfo-Quinolin-8-Yloxy) Phthalate and Bipyridinyl Coligands: Structure Diversity and Properties. J. Solid State Chem..

[19] Kumar K., Godeto T., Darkwa J. (2016). Tandem Ethylene Dimerization and Friedel-Crafts Alkylation of Toluene Catalyzed by Homo- and Heterogeneous Nickel(II) and Palladium(II) Pre-Catalysts. J. Organomet. Chem..

[20] Griffith G.W. (1984). Quantitation of Silanol in Silicones by FTIR Spectroscopy. Ind. Eng. Chem. Prod. Res. Dev..

[21] da Silva F.P., Rossi L.M. (2014). Palladium on Magnetite: Magnetically Recoverable Catalyst for Selective Hydrogenation of Acetylenic to Olefinic Compounds. Tetrahedron.

[22] Jin X., Zhang K., Sun J., Wang J., Dong Z., Li R. (2012). Magnetite Nanoparticles Immobilized Salen Pd (II) as a Green Catalyst for Suzuki Reaction. Catal. Commun..

[23] GHORBANI-VAGHEI R., HEMMATI S., HEKMATI M. (2016). Pd Immobilized on Modified Magnetic Fe_3_O_4_ Nanoparticles: Magnetically Recoverable and Reusable Pd Nanocatalyst for Suzuki-Miyaura Coupling Reactions and Ullmann-Type N-Arylation of Indoles. J. Chem. Sci..

[24] Halligudra G., Paramesh C.C., Mudike R., Ningegowda M., Rangappa D., Shivaramu P.D. (2021). PdII on Guanidine-Functionalized Fe_3_O_4_ Nanoparticles as an Efficient Heterogeneous Catalyst for Suzuki–Miyaura Cross-Coupling and Reduction of Nitroarenes in Aqueous Media. ACS Omega.

[25] Lara L.R.S., Zottis A.D., Elias W.C., Faggion D., Maduro de Campos C.E., Acuña J.J.S., Domingos J.B. (2015). The Catalytic Evaluation of in Situ Grown Pd Nanoparticles on the Surface of Fe_3_O_4_@dextran Particles in the P-Nitrophenol Reduction Reaction. RSC Adv..

[26] Shard A.G. (2020). Practical Guides for X-ray Photoelectron Spectroscopy: Quantitative XPS. J. Vac. Sci. Technol. A.

[27] Biesinger M.C., Hart B.R., Polack R., Kobe B.A., Smart R.S.C. (2007). Analysis of Mineral Surface Chemistry in Flotation Separation Using Imaging XPS. Miner. Eng..

[28] Liang H.-W., Wei W., Wu Z.-S., Feng X., Müllen K. (2013). Mesoporous Metal–Nitrogen-Doped Carbon Electrocatalysts for Highly Efficient Oxygen Reduction Reaction. J. Am. Chem. Soc..

[29] Casanovas J., Ricart J.M., Rubio J., Illas F., Jiménez-Mateos J.M. (1996). Origin of the Large N 1s Binding Energy in X-ray Photoelectron Spectra of Calcined Carbonaceous Materials. J. Am. Chem. Soc..

[30] Zhao P., Nie H., Yu J., Wang J., Cheng G. (2018). A Facile Synthesis of Porous N-Doped Carbon with Hybridization of Fe3C Nanoparticle-Encased CNTs for an Advanced Oxygen Reduction Reaction Electrocatalyst. Inorg. Chem. Front..

[31] Yamashita T., Hayes P. (2008). Analysis of XPS Spectra of Fe^2+^ and Fe^3+^ Ions in Oxide Materials. Appl. Surf. Sci..

[32] Zhang Y.C., Tang J.Y., Hu X.Y. (2008). Controllable Synthesis and Magnetic Properties of Pure Hematite and Maghemite Nanocrystals from a Molecular Precursor. J. Alloys Compd..

[33] He H., Zhong Y., Liang X., Tan W., Zhu J., Wang C.Y. (2015). Natural Magnetite: An Efficient Catalyst for the Degradation of Organic Contaminant. Sci. Rep..

[34] Wang W., Xie Y., Zhang S., Liu X., Haruta M., Huang J. (2018). Selective Hydrogenation of Cinnamaldehyde Catalyzed by ZnO-Fe_2_O_3_ Mixed Oxide Supported Gold Nanocatalysts. Catalysts.

[35] Chen Y.Z., Wei S.W., Wu K.J. (1993). Effect of Promoter on Selective Hydrogenation of α,β-Unsaturated Aldehydes over Cobalt Borides. Appl. Catal. A Gen..

[36] Ponec V. (1997). On the Role of Promoters in Hydrogenations on Metals; α, β-Unsaturated Aldehydes and Ketones. Appl. Catal. A Gen..

[37] Patel A., Patel K. (2014). Tetranuclear Mn(II) Substituted Sandwich Complex as a Recyclable Homogeneous Catalyst for Selective Oxidation of Styrene to Benzaldehyde: Effect of Reaction Parameters and Kinetics. Inorg. Chim. Acta.

[38] Chernyshev V.M., Astakhov A.V., Chikunov I.E., Tyurin R.V., Eremin D.B., Ranny G.S., Khrustalev V.N., Ananikov V.P. (2019). Pd and Pt Catalyst Poisoning in the Study of Reaction Mechanisms: What Does the Mercury Test Mean for Catalysis?. ACS Catal..

[39] Oklu N.K., Makhubela B.C.E. (2018). Highly Selective and Efficient Solvent-Free Transformation of Bio-Derived Levulinic Acid to γ-Valerolactone by Ru (II) Arene Catalyst Precursors. Inorg. Chim. Acta.

[40] Gruber-Woelfler H., Radaschitz P.F., Feenstra P.W., Haas W., Khinast J.G. (2012). Synthesis, Catalytic Activity, and Leaching Studies of a Heterogeneous Pd-Catalyst Including an Immobilized Bis(Oxazoline) Ligand. J. Catal..

[41] Kresse G., Hafner J. (1993). Ab Initio Molecular Dynamics for Liquid Metals. Phys. Rev. B Condens. Matter.

[42] Kresse G., Hafner J. (1994). Ab Initio Molecular-Dynamics Simulation of the Liquid-Metal-Amorphous-Semiconductor Transition in Germanium. Phys. Rev. B Condens. Matter.

[43] Kresse G., Furthmüller J. (1996). Efficient Iterative Schemes for Ab Initio Total-Energy Calculations Using a Plane-Wave Basis Set. Phys. Rev. B Condens. Matter.

[44] Perdew J.P., Burke K., Ernzerhof M. (1996). Generalized Gradient Approximation Made Simple. Phys. Rev. Lett..

[45] Dudarev S.L., Botton G.A., Savrasov S.Y., Humphreys C.J., Sutton A.P. (1998). Electron-Energy-Loss Spectra and the Structural Stability of Nickel Oxide: An LSDA+U Study. Phys. Rev. B.

[46] Anisimov V.I.V.I., Korotin M.A., Zaanen J., Andersen O.K. (1992). Spin Bags, Polarons, and Impurity Potentials in La_2_-XSrxCuO_4_ from First Principles. Phys. Rev. Lett..

[47] Santos-Carballal D., Roldan A., Grau-Crespo R., de Leeuw N.H. (2014). A DFT Study of the Structures, Stabilities and Redox Behaviour of the Major Surfaces of Magnetite Fe_3_O_4_. Phys. Chem. Chem. Phys..

[48] Grimme S., Antony J., Ehrlich S., Krieg H. (2010). A Consistent and Accurate Ab Initio Parametrization of Density Functional Dispersion Correction (DFT-D) for the 94 Elements H-Pu. J. Chem. Phys..

[49] Monkhorst H.J., Pack J.D. (1976). Special Points for Brillouin-Zone Integrations. Phys. Rev. B.

