# Supplementary material for: The Transfer Hydrogenation of Cinnamaldehyde Using Homogeneous Cobalt(II) and Nickel(II) (E)-1-(Pyridin-2-yl)-N-(3-(triethoxysilyl)propyl)methanimine and the Complexes Anchored on Fe3O4 Support as Pre-Catalysts: An Experimental and In Silico Approach
Source: Molecules. 2023 Jan 9;28(2):659. doi: 10.3390/molecules28020659 (PMC9865650; doi:10.3390/molecules28020659)
Supplement: Supplementary file 1 [file molecules-28-00659-s001.zip › molecules-2067952-supplementary.pdf]

The Transfer Hydrogenation of Cinnamaldehyde Using Homogeneous Cobalt(II) and Nickel(II) (E)-1-(Pyridin-2-yl)-N-(3-(triethoxysilyl)propyl)methanimine and the Complexes Anchored on Fe_3O_4 Support as Pre-Catalysts: An Experimental and In Silico Approach

Fortunate P. Sejie [1], Olayinka A. Oyetunji [1,*], James Darkwa [2,3,*], Isaac N. Beas [2], Banothile C. E. Makhubela [3], Nelson Y. Dzade [4,5] and Nora H. de Leeuw [5,6,*]

[1] Department of Chemistry, University of Botswana, Gaborone Private Bag UB 00704, Botswana
[2] Botswana Institute for Technology Research and Innovation, Gaborone Private Bag 0082, Botswana
[3] Department of Chemical Sciences, University of Johannesburg, P.O. Box 524, Auckland Park 2006, South Africa
[4] Department of Energy and Mineral Engineering, Pennsylvania State University, University Park, PA 16802, USA
[5] School of Chemistry, Cardiff University, Main Building, Park Place, Cardiff CF10 3AT, UK
[6] School of Chemistry, University of Leeds, Leeds LS2 9JT, UK
* Correspondence: oyetunji@ub.ac.bw (O.A.O.); jdarkwa@bitri.co.bw (J.D.); n.h.deleeuw@leeds.ac.uk (N.H.d.L.)

Supplementary material

Synthesis of magnetite

Superparamagnetic iron oxide (Fe_3O_4) nanoparticles were prepared according to the previously reported procedure with some modifications. A mixture of $FeCl_3.6H_2O$ (2.6 g) and $FeCl_2.4H_2O$ (1.0 g) was dissolved in deoxygenated milli-Q water (12.5 mL) and 10 M HCl (0.425 mL) with sonication. The resulting solution was added dropwise into 125 mL of 1.5 M NH_3 under vigorous stirring. This led to the formation of a brown precipitate which was isolated using a magnet. The magnetite particles were separated by an external magnet and washed with water to remove excess ammonia.

Immobilisation of catalysts on magnetite

The immobilized catalyst were synthesized by a modification of method reported in the literature. with modification. The catalysts, M@Fe_3O_4 were prepared by dispersing 0.3 g of Fe_3O_4 was in 30mL of toluene (30 mL) by ultra-sonication for 0.5 h. The metal precursor Nickel diethylene glycol bis bromo (2 mmol) was added to the reaction mixture and the reaction mixture was centrifuged, and the solvent discarded. The solid product was washed with deionized water and dried in air.

3.2 (E)-1-(pyridin-2-yl)-N-(3-(triethoxysilyl)propyl)methanimino Nickel bromide (1)

The synthesis of **C2** was performed in a similar manner as described in the literature[30]. Melting point:182-186°C. FTIR Shimazu 2971cm^{-1}(ν_{C-N}), 1646cm^{-}($\nu_{C=N}$) ,1446cm^{-1}(ν_{C-C-O}), 1390cm^{-1}(ν_{C-C}), 1164cm^{-1}(ν_{C-C}),1069cm^{-1}(ν_{Si-O}), 953cm^{-1}(ν_{O-C}). Elemental Analysis (found values are in parenthesis): C, 34.06(33.71); H, 4.95(4.89); N, 5.30(5.28); Mass spectroscopy: ESI$^+$ *m/z* 614= $[C_{16}H_{29}Br_2C_{12}N_2NiO_3Si]^+$, m/z 263= $[C_{15}H_{22}Br_2N_2NiO_3Si]^{+2}$

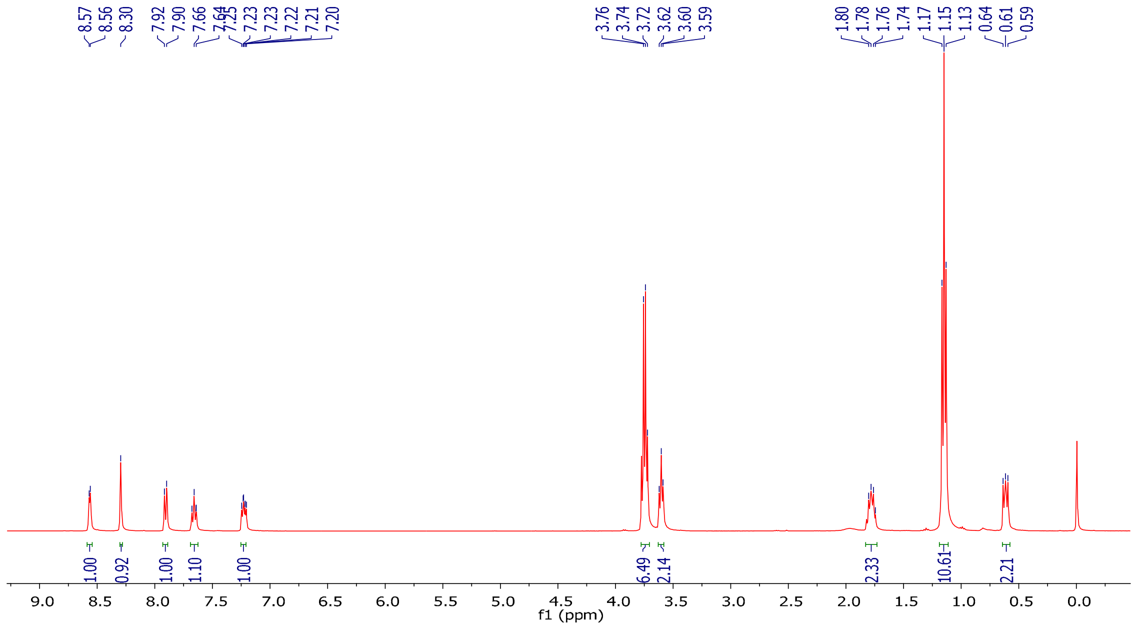

Figure S1.^{1}HNMR spectra of (E)-1-(pyridin-2-yl)-N-(3-(triethoxysilyl)propyl)methanimine.

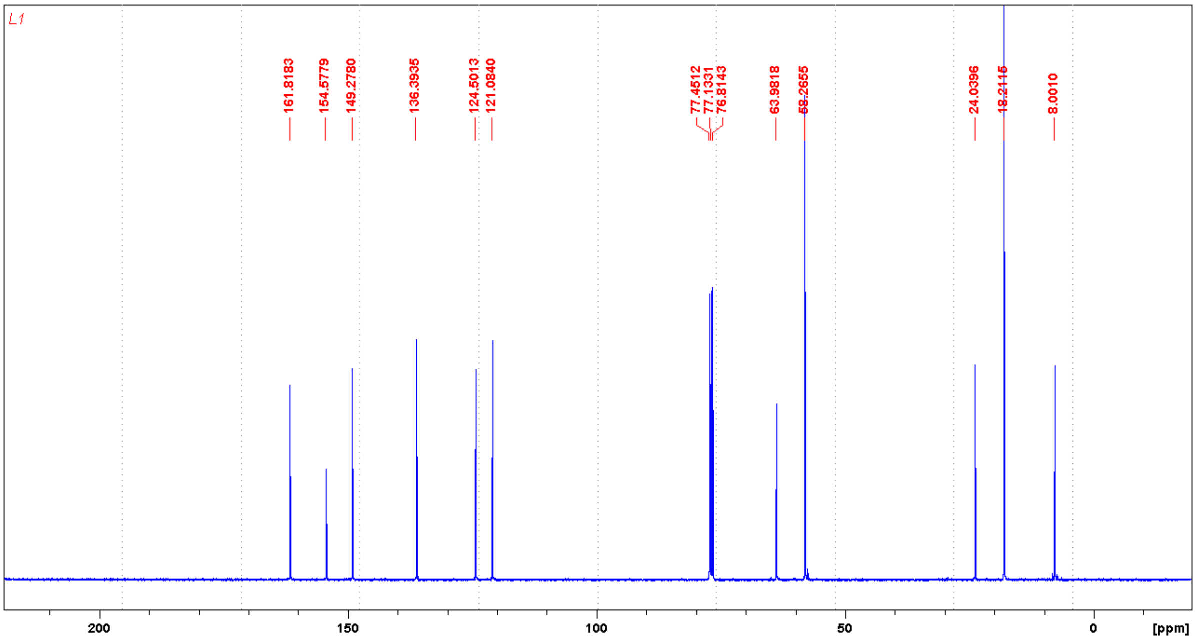

Figure S2. The ^{13}C NMR spectra of 2:(E)-1-(pyridin-2-yl)-N-(3-(triethoxysilyl)propyl)methanimine.

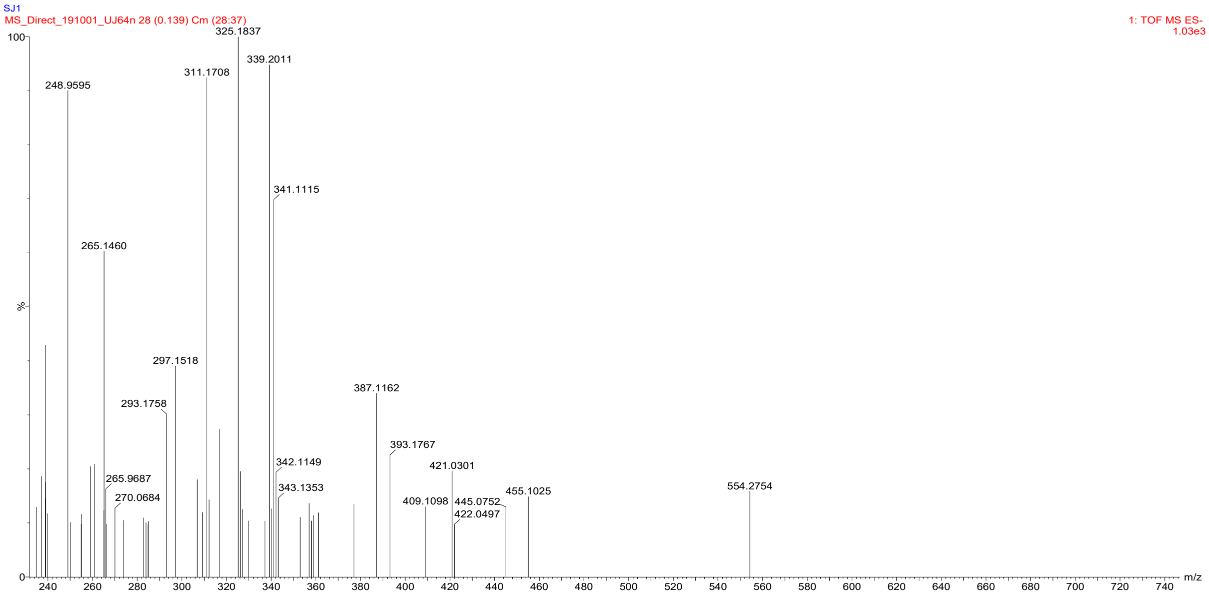

Figure S3. Time of Flight electro spray ionisation spectra of **C2** in negative mode.

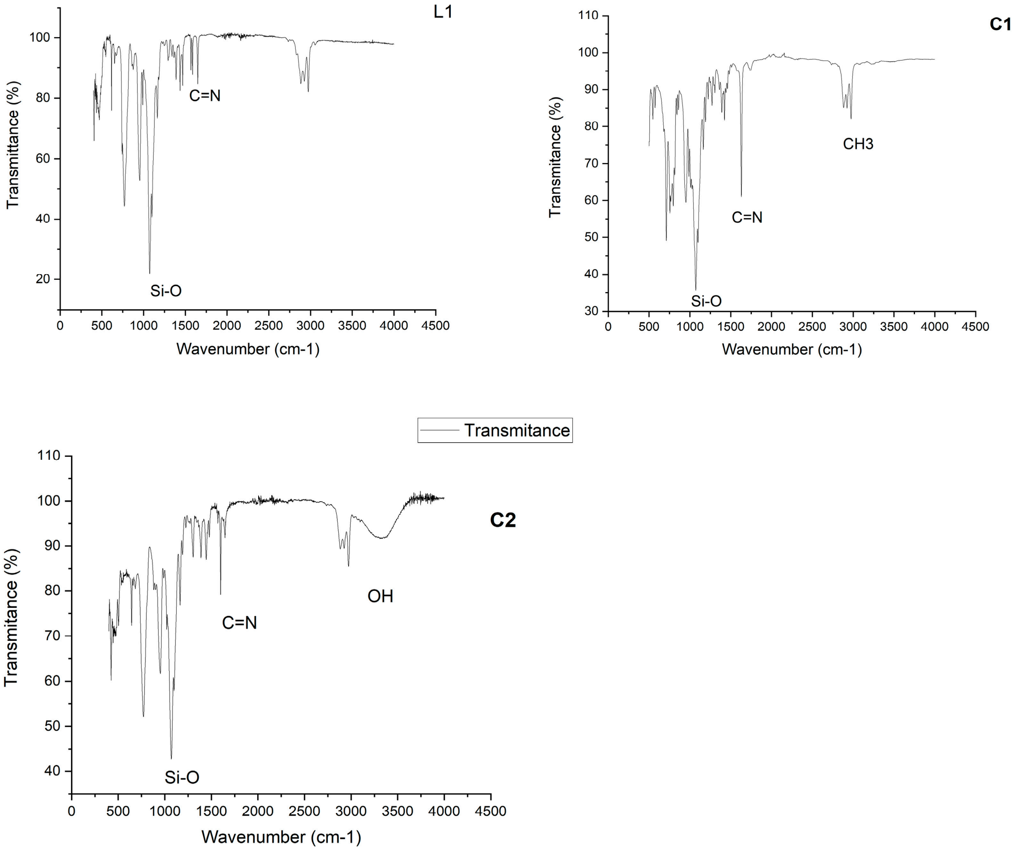

Figure S4. The IR spectra of the ligand and the corresponding homogeneous catalysts.

Synthesis of :(E)-1-(pyridin-2-yl)-N-(3-(triethoxysilyl)propyl)methanimine functionalized Fe_3O_4

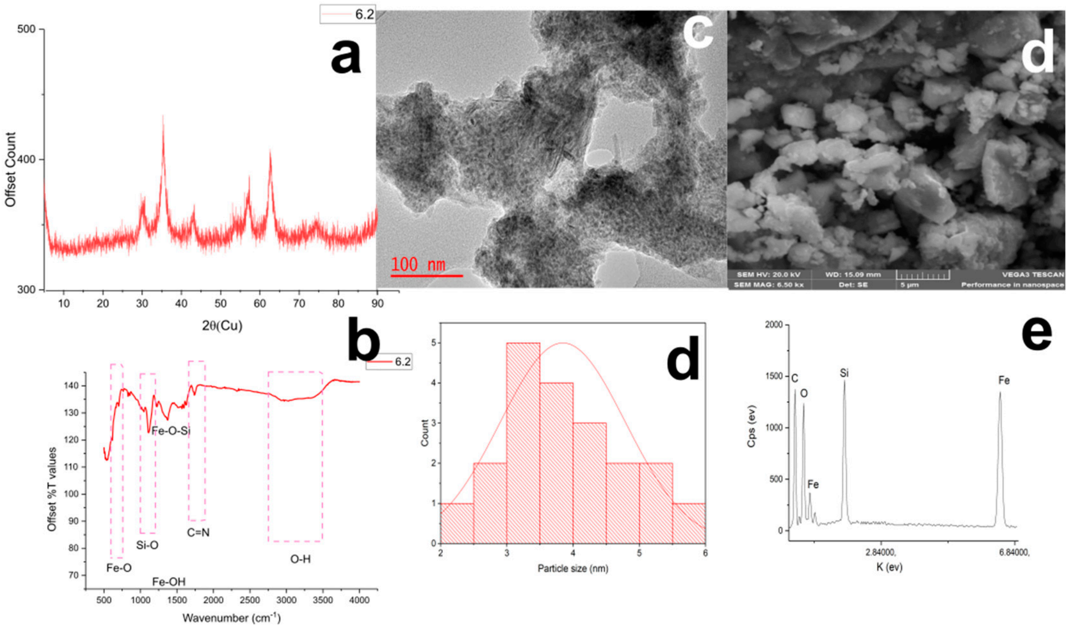

Figure S5. P-XRD, FTIR, SEM, EDS, particle size distribution and TEM characterization of Fe3O4-immino-py.

Table S1. Calculated strain on the lattice of magnetite for the supported pre-catalysts, ligand, and the support.

**Catalyst/compound**	**Metal load(m/m) %**	**Calculated microstrain(εx10^{-3})**
Magnetite	0	0
**Fe_3O_4**-immino-py	0	9.8
**Fe_3O_4**@C2	1.37	7.0
**Fe_3O_4**@C1	1.22	6.9

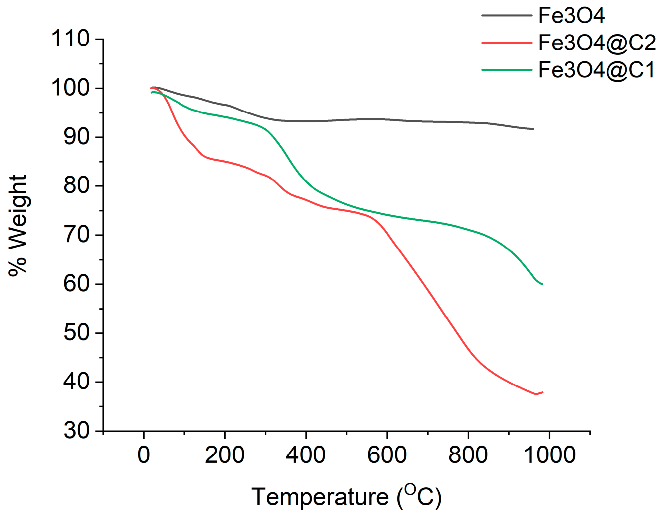

Figure S6. Thermal gravimetric analysis of the supported pre-catalysts.

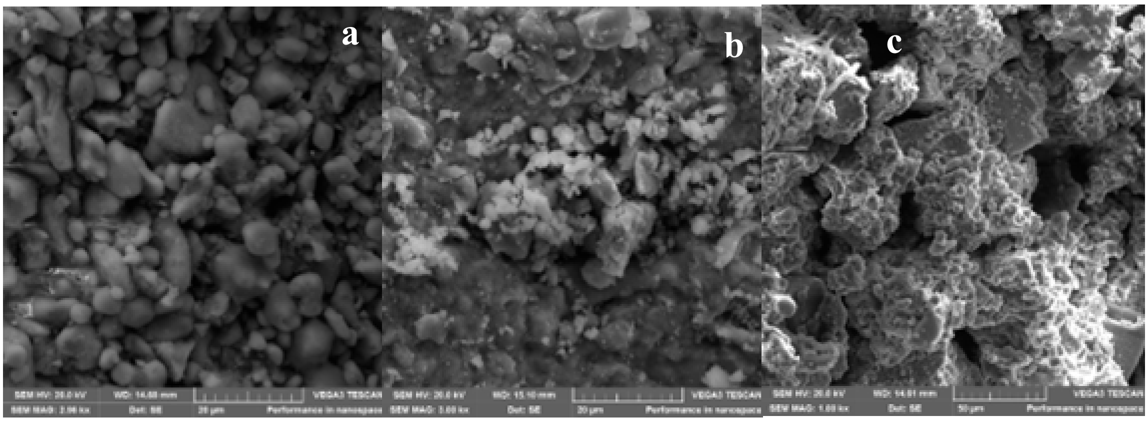

Figure S7. The SEM images of the fresh heterogeneous catalysts.

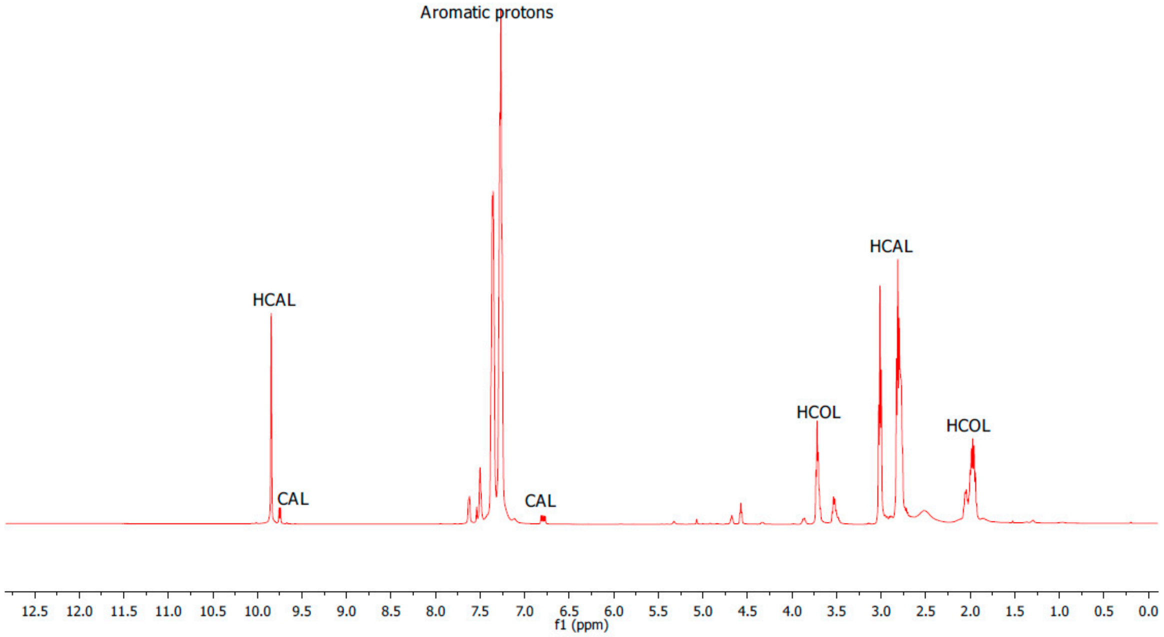

Figure S8. Example of the H NMR spectra of the isolated products of CAL hydrogenation using homogeneous **C2** catalyst after 16h of reaction time.

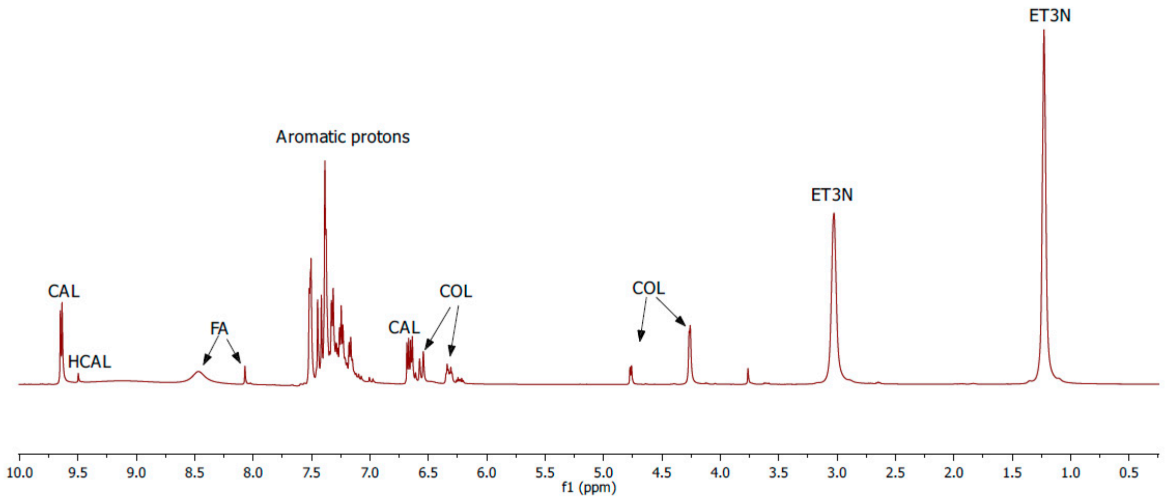

Figure S9. Example of the H NMR spectra of the crude mixture after CAL hydrogenation using ***Fe₃O₄@C2*** after catalyst 8h of reaction time.

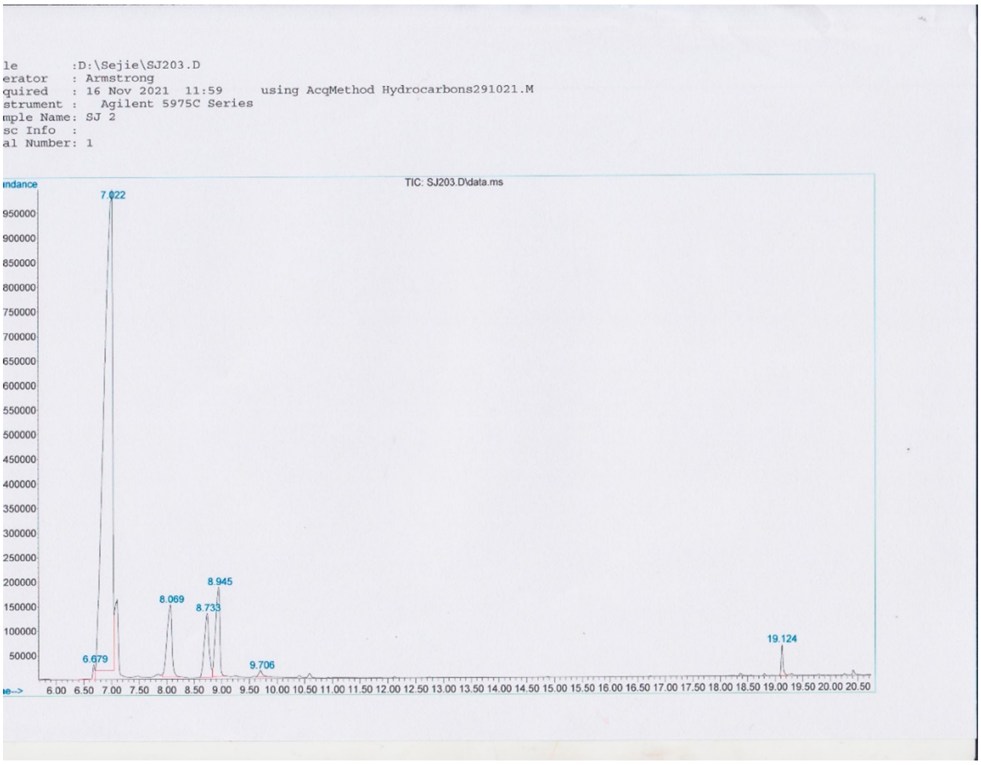

Figure S10. Example of the GC spectra of CAL hydrogenation using homogeneous **C2** catalyst.

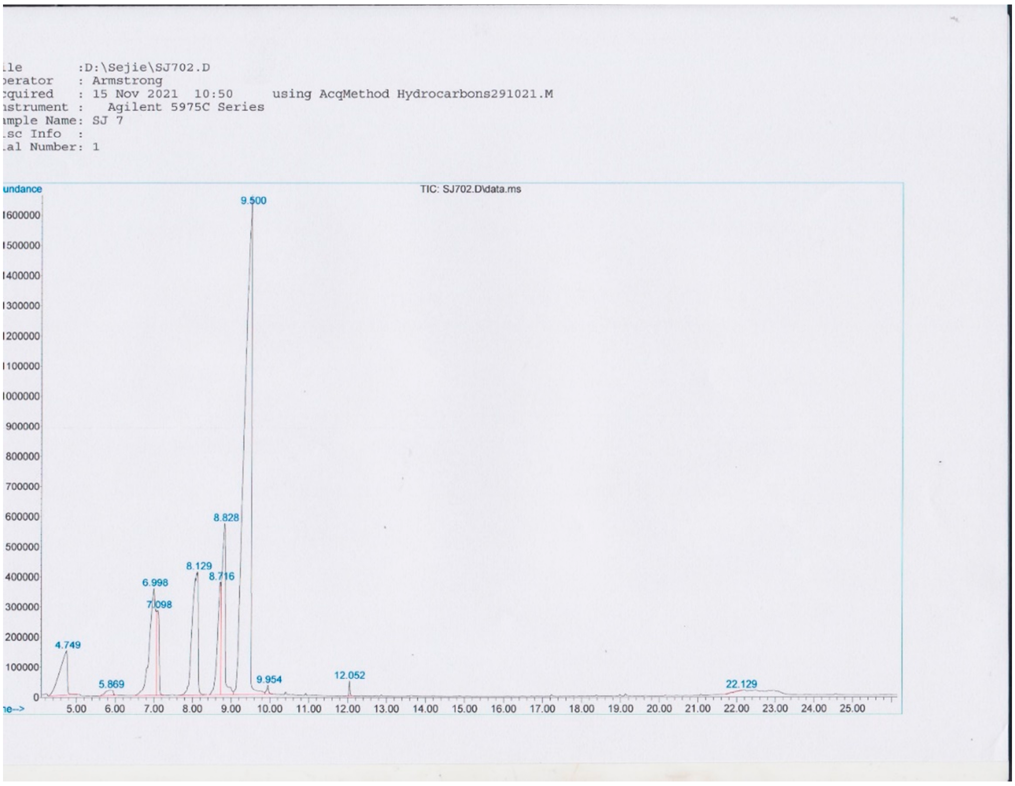

Figure S11. Example of the GC spectra of CAL hydrogenation using homogeneous **Fe3O4@C2** catalyst.

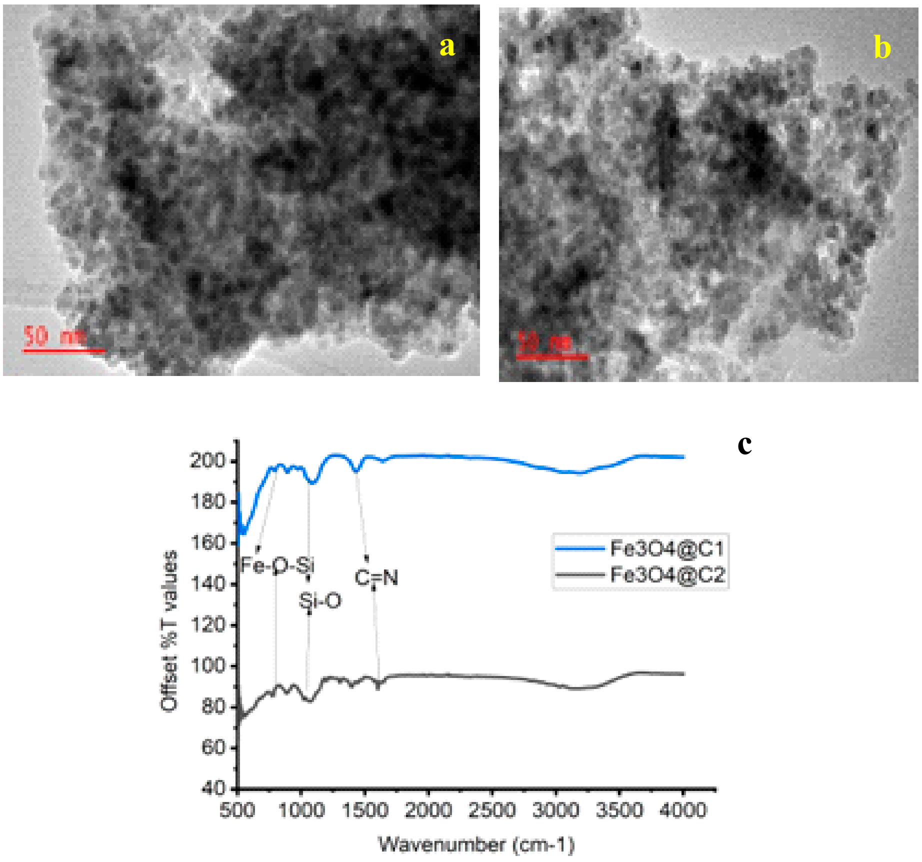

Figure S12. The TEM images of catalyst ***Fe3O4*@C2** (a) and ***Fe3O4*@C1**(b) and the IR analysis(c) of after the 9th cycle.

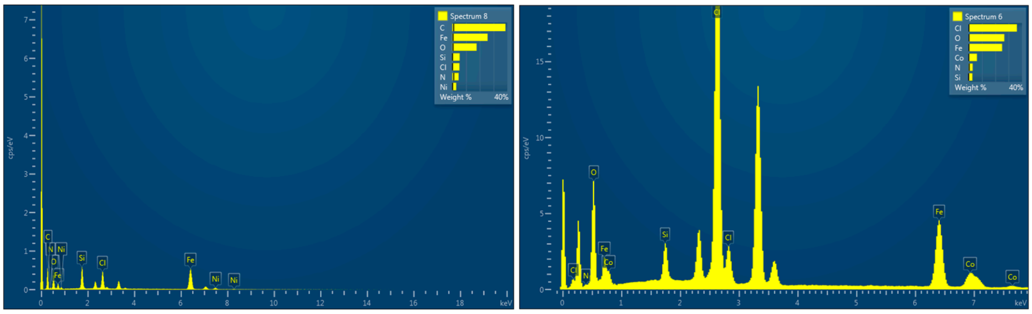

Figure S13. The EDS spectra of the spent catalysts.

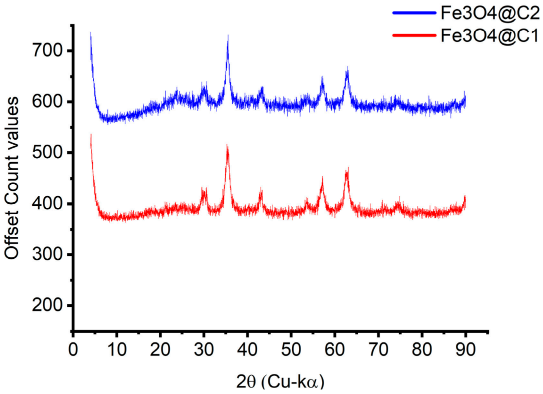

Figure S14. The XRD of the spent catalysts.

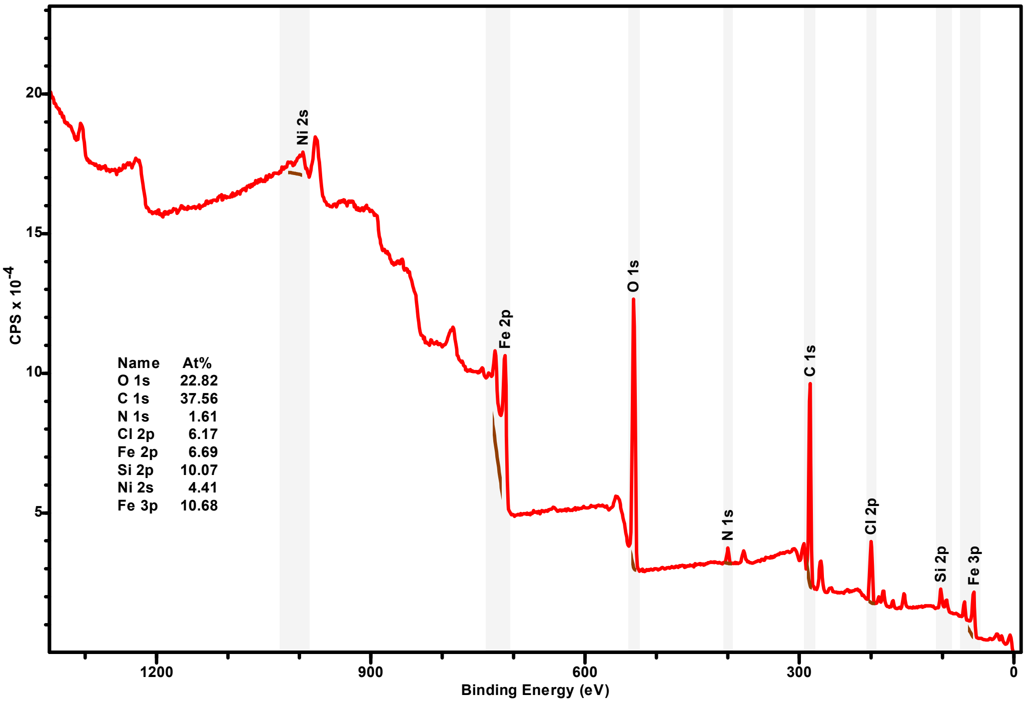

Figure S15. The XPS survey of spent **Fe_3O_4@C2**.

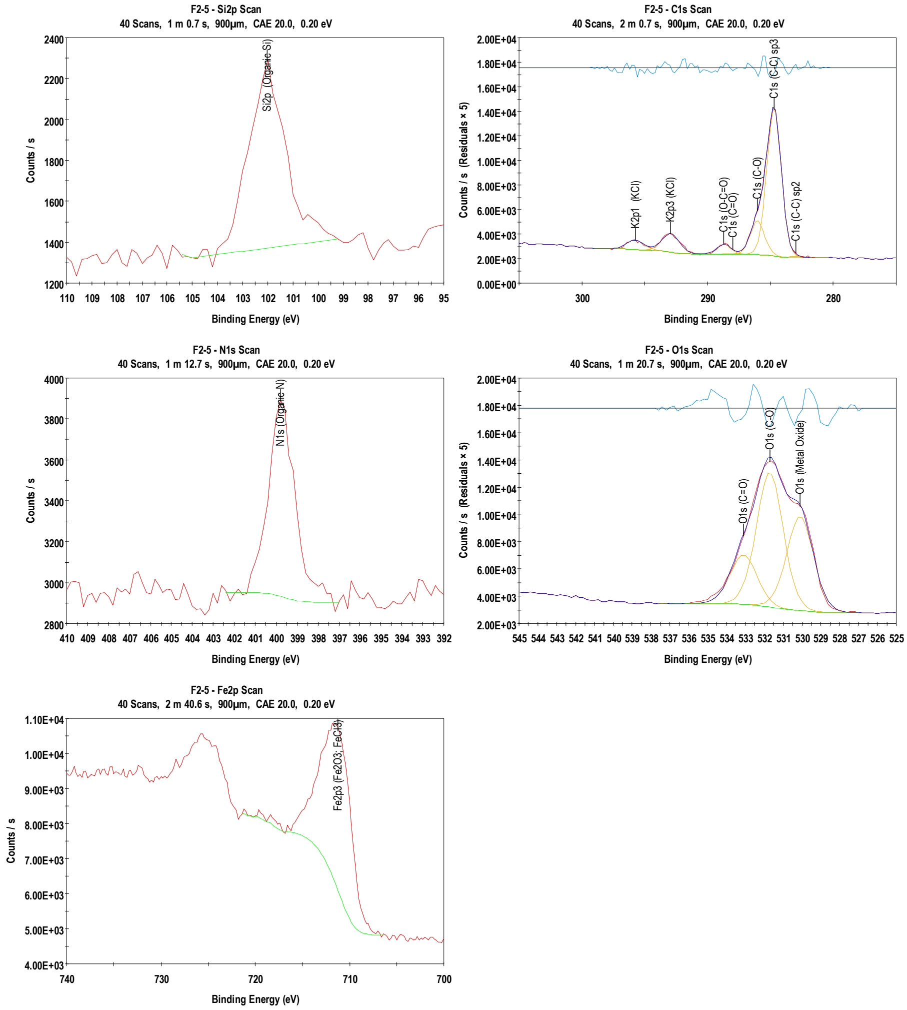

Figure S16. The high-resolution analysis of the elements detected in **Fe_3O_4@C2.**

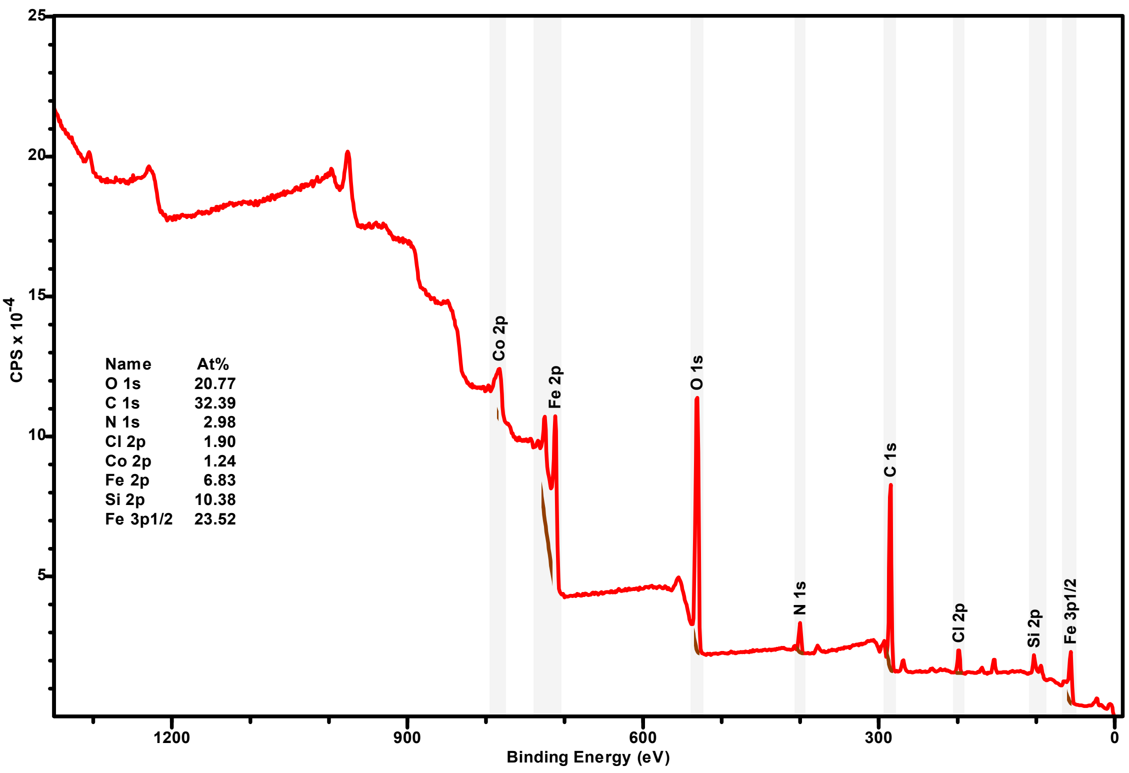

Figure S17. The XPS survey of spent **Fe_3O_4@C1**.

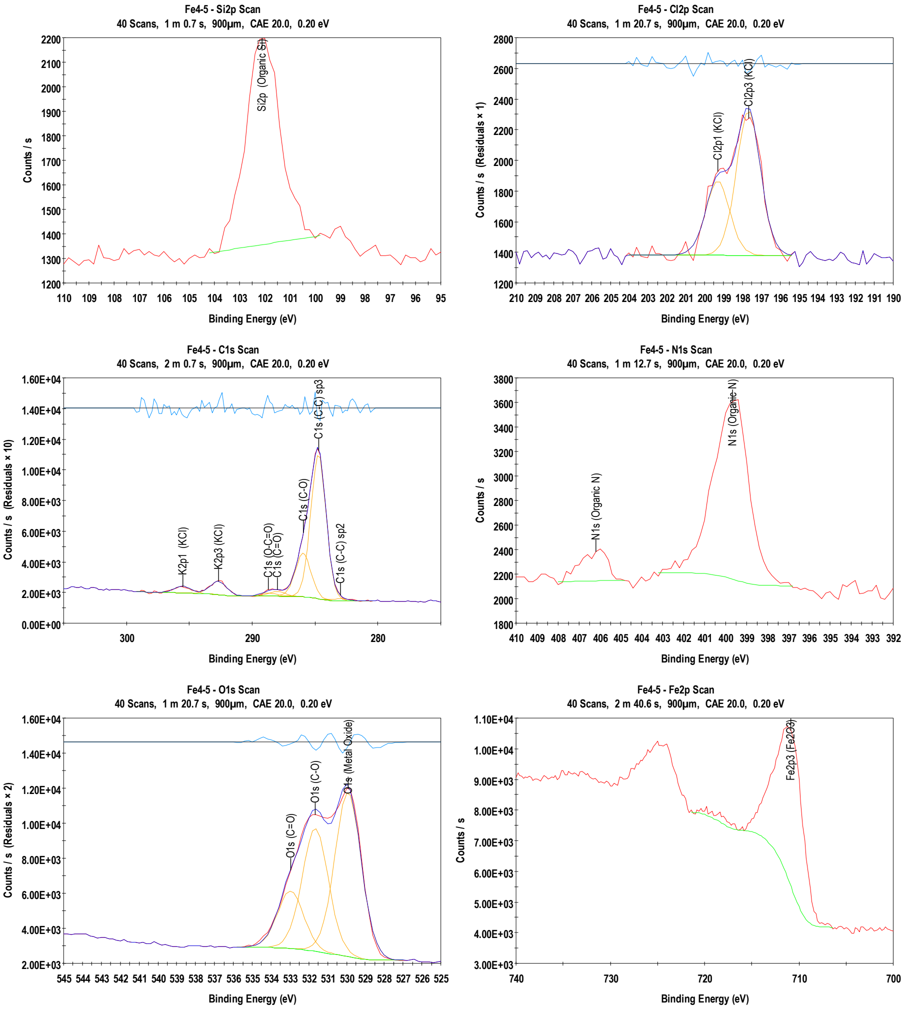

Figure S18. The high-resolution analysis of the elements detected in **Fe_3O_4@C1.**

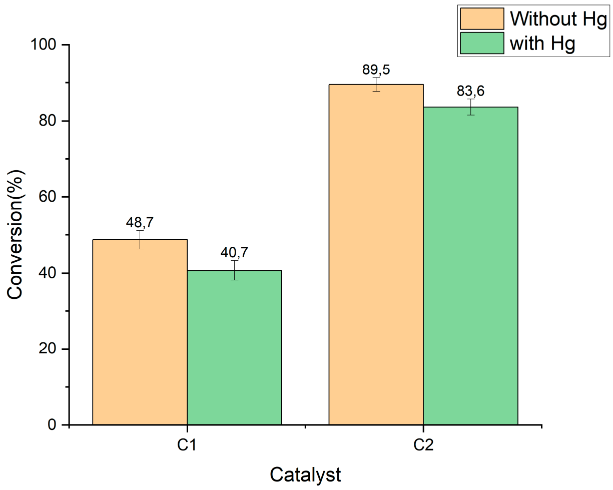

Figure S19. Homogeneity tests for the transfer hydrogenation of CAL using catalysts **C1** and **C2** in the presence of liquid mercury . Reaction conditions: CAL (20 mmol), catalyst (0.02 mmol) formic acid (40mmol), base (20 mmol), 120 ^{o}C, 2 mg Hg (0), 24 h. Conversions are estimated by gas chromatography.

Figure S1:^{1}HNMR spectra of (E)-1-(pyridin-2-yl)-N-(3-(triethoxysilyl)propyl)methanimine 2
Figure S2 The ^{13}C NMR spectra of 2:(E)-1-(pyridin-2-yl)-N-(3-(triethoxysilyl)propyl)methanimine 2
Figure S3 Time of Flight electro spray ionisation spectra of **C2** in negative mode 3
Figure S4 The IR spectra of the ligand and the corresponding homogeneous catalysts 3
Figure S5: P-XRD, FTIR, SEM, EDS, particle size distribution and TEM characterization of Fe3O4-immino-py 4
Figure S6: Thermal gravimetric analysis of the supported pre-catalysts 4
Figure S7 The SEM images of the fresh heterogeneous catalysts 5
Figure S8 :Example of the H NMR spectra of the isolated products of CAL hydrogenation using homogeneous **C2** catalyst after 16h of reaction time 5
Figure S9: Example of the H NMR spectra of the crude mixture after CAL hydrogenation using **Fe3O4@C2** after catalyst 8h of reaction time 6
Figure S10 :Example of the GC spectra of CAL hydrogenation using homogeneous **C2** catalyst. 6
Figure S11 :Example of the GC spectra of CAL hydrogenation using homogeneous **Fe3O4@C2** catalyst. 7
Figure S12 The TEM images of catalyst **Fe_3O_4@C2** (a) and **Fe_3O_4@C1**(b) and the IR analysis(c) of after the 9th cycle7
Figure S13The EDS spectra of the spent catalysts...8
Figure S14 The XRD of the spent catalysts 8
Figure S 15 The XPS survey of spent **Fe_3O_4@C2**...8
Figure S 16 The high-resolution analysis of the elements detected in **Fe_3O_4@C2**..9
Figure S 17 The XPS survey of spent **Fe_3O_4@C1**...10
Figure S 18 The high-resolution analysis of the elements detected in **Fe_3O_4@C1**..11
Figure S19**:** Homogeneity tests for the transfer hydrogenation of CAL using catalysts **C1** and **C2** in the presence of liquid mercury . Reaction conditions: CAL (20 mmol), catalyst (0.02 mmol) formic acid (40mmol), base (20 mmol), 120 ^{o}C, 2 mg Hg (0), 24 h. Conversions are estimated by gas chromatography. 11
